# Enhanced detection of distinct honeycomb-structured neuronal SMARCC2 cytobodies in Parkinson’s Disease via Cyclic Heat-Induced Epitope Retrieval (CHIER)

**DOI:** 10.1371/journal.pone.0315183

**Published:** 2024-12-17

**Authors:** Amber Carmichael-Lowe, Brionne Fleming, Kreesan Reddy, James Wiseman, Eden Paige Yin, Clinton P. Turner, Richard L. M. Faull, Maurice A. Curtis, Mike Dragunow, Birger Victor Dieriks

**Affiliations:** 1 Department of Anatomy and Medical Imaging, University of Auckland, Auckland, New Zealand; 2 Centre for Brain Research, University of Auckland, Auckland, New Zealand; 3 Anatomical Pathology, Pathology and Laboratory Medicine, Auckland City Hospital, Auckland, New Zealand; 4 Department of Pharmacology, University of Auckland, Auckland, New Zealand; Università di Pisa: Universita degli Studi di Pisa, ITALY

## Abstract

Antigen retrieval is crucial for immunohistochemistry, particularly in formalin-fixed paraffin-embedded brain tissue, where fixation causes extensive crosslinking that masks epitopes. Heat Induced Epitope Retrieval (HIER) reverses these crosslinks, improving access to nuclear and aggregated proteins. We introduce Cyclic Heat-Induced Epitope Retrieval (CHIER), an advanced technique that builds on HIER by incorporating repeated cycles of heating and cooling. CHIER optimises antigen retrieval and significantly improves detection. CHIER is particularly effective for detecting chromatin-binding proteins, such as SMARCC2, which are difficult to label using conventional IHC methods. Using CHIER on formalin-fixed paraffin-embedded human brain sections, we achieved robust detection of SMARCC2 in both the nucleus and cytoplasm. CHIER also enhanced the visualisation of large SMARCC2+ cytoplasmic bodies, termed cytobodies, which are increased in Parkinson’s Disease (PD). Our findings suggest that SMARCC2 may translocate from the nucleus to the cytoplasm in PD, potentially implicating SMARCC2 aggregation in the disease’s pathology. Furthermore, CHIER does not negatively impact the antigenicity of other antibodies, supporting its use for multiplex fluorescent immunohistochemistry and super-resolution imaging. These results highlight CHIER’s potential for improving the detection of chromatin-binding and aggregated proteins in neurodegenerative disease research, offering new insights into SMARCC2’s role in Parkinson’s Disease.

## Introduction

Antigen retrieval is crucial in immunohistochemistry (IHC), particularly for formalin-fixed paraffin-embedded (FFPE) human brain sections. These tissues undergo extensive crosslinking during the fixation process, which masks antigenic sites and prevents effective antibody binding. Heat Induced Epitope Retrieval (HIER) is frequently used to reverse these crosslinks, improving the exposure of linear epitopes, especially in nuclear and cytoplasmic proteins [1].

Hydrated heating during HIER is particularly effective for unmasking antigens hidden within tightly packed chromatin structures. For example, nuclear antigens such as p53 and Ki67 and cytoskeletal and membrane proteins often benefit from this technique. The HIER process likely disrupts formalin-induced protein cross-links and destabilises DNA, converting double-stranded DNA into single strands and enabling antibodies to access previously masked epitopes [2, 3]. Formic acid treatment is often employed in addition to HIER to improve aggregated protein detection [4, 5].

Cyclic Heat-Induced Epitope Retrieval (CHIER) builds upon the HIER method by incorporating repeated cycles of dry heating and cooling preceding the standard epitope retrieval procedure. CHIER is an additional antigen retrieval treatment whereby tissue sections undergo several cycles of timed heating and cooling on a hot plate. We hypothesised that it primarily benefits chromatin-associated and/or aggregated proteins like SMARCC2, whose antigenic sites are often buried within nucleic acid stretches.

SWI/SNF Related, Matrix Associated, Actin Dependent Regulator of Chromatin Subfamily C Member 2 (SMARCC2), also known as Mammalian Chromatin Remodelling Complex BRG1-Associated Factor 170 (Baf170), is a core subunit of the SWI/SNF family chromatin remodelling complexes. The Switch/Sucrose Nonfermentable (SWI/SNF) complex plays a crucial role in chromatin remodelling and the regulation of transcription by recruiting transcription factors, coactivators, repressors, and histone modifiers [6–8]. These multi-subunit, ATP-dependent molecular machines slide and evict nucleosomes [8]. These elements are vital at various stages of neurogenesis in both postnatal and adult stages. When chromatin regulation is disrupted, it can lead to faults in epigenetic gene regulation and result in abnormal gene expression patterns. These irregularities also contribute to a variety of chronic pathologies, including neurodegenerative diseases like Parkinson’s Disease (PD) [9].

When we apply CHIER for SMARCC2 antibody detection, we show that it enhances epitope retrieval, which is difficult to label with conventional methods. By implementing CHIER, we have successfully detected SMARCC2 in the nucleus and improved the detection of large SMARCC2^+^ cytoplasmic bodies called cytobodies in human brain tissue. We subsequently investigated the potential role of SMARCC2 further and found that SMARCC2 can translocate from the nucleus to the cytoplasm. Our results show that these cytobodies are increased in PD and potentially implicate SMARCC2 aggregation in the pathological process of PD.

## Materials and methods

### Human brain tissue

Human post-mortem brain tissue was received from the Neurological Foundation Human Brain Bank at the Centre for Brain Research, University of Auckland, New Zealand. All brain tissue was donated with written informed consent from donors and their families. All protocols followed relevant guidelines and regulations approved by the University of Auckland Human Participants Ethics Committee (Ref: 011654–14/NTA/208). Brain tissue specimens was first accessed for research purposes on 27/05/2019. A neuropathologist assessed all cases used in this study. The neurologically normal cases (*n* control = 22) had no clinical history of neurological abnormalities, and no other significant neuropathology was noted upon post-mortem examination. The mean age (± SD) of control cases was 70 ± 17, ranging from 35 to 98 years (Table 1). The mean post-mortem delay (PMD) of neurologically normal control cases was 17 ± 7 hours with a range of 4–33 hours. All PD cases (*n* = 22) had a clinical history of PD, and pathological features were consistent with PD pathology, as confirmed by a neuropathologist. Key neuropathological features were loss of pigment and pigmented cells in the substantia nigra and accumulation of LBs in the substantia nigra and other brain regions; many cases also had evidence of cortical LB disease. PD cases had a disease duration ranging from 1–25 years; the mean duration was 14 ± 7 years (Table 1). The mean age of PD cases was 78 ± 8 and ranged from 60–91 years; the mean post-mortem delay was 11 ± 7 hours with a range of 2.25–25 hours.

**Table 1:** Case information for post-mortem middle temporal gyrus brain tissue (MTG) used in this study.

Case	age (years)	Sex	PMD (hours)	brain weight (g)	cause of death	duration of PD (years)
**H180**	73	M	33	1318	Ischaemic heart disease	
**H184**	35	M	20	1594	Electrocution	
**H186**	68	M	21	1327	Ischaemic heart disease	
**H187**	98	F	15	1114	Caecal carcinoma	
**H189**	41	M	16	1412	Asphyxia	
**H204**	66	M	9	1461	Ischaemic heart disease	
**H209**	48	M	23	1470	Ischaemic heart disease	
**H226**	65	M	8	1279	Ischaemic heart disease	
**H227**	78	F	4	971	cerebrovascular accident	
**H228**	87	F	21	1376	Ischaemic heart disease	
**H231**	65	M	8	1527	Ischaemic heart disease	
**H237**	81	M	17	1267	acute bacterial endocarditis	
**H238**	63	F	16	1324	Dissecting aortic aneurysm	
**H239**	64	M	15.5	1529	Ischemic Heart Disease	
**H242**	61	M	19.5	1466	Coronary atherosclerosis	
**H243**	77	F	13	1184	Ischaemic heart disease	
**H244**	76	M	16	1508	Ischaemic heart disease	
**H245**	63	M	20	1194	Asphyxia	
**H246**	89	M	17	1130.7	Type II myocardial infarction	
**H247**	51	M	31	1671	Bilateral pulmonary thromboembolism	
**H250**	93	F	19	1143.3	Acute coronary syndrome; pneumonia	
**H253**	98	F	15	1147	Caecal carcinoma	
**PD30**	82	M	19	1222	Multiple organ failure	11
**PD31**	67	M	25	1405	Respiratory failure	5
**PD32**	71	M	8	1490	Ischaemic heart disease	25
**PD35**	73	M	16	1293	Pneumonia	15
**PD37**	81	M	4	1190	PD	13
**PD41**	81	M	13	1248	Renal failure/urinary sepsis	1
**PD43**	60	F	15.5	1118	Bronchopneumonia & multisystem organ failure	7
**PD50**	88	M	6	1218	ischaemic heart disease	20
**PD52**	84	M	5	1067	Myocardial infarction	12
**PD56**	74	M	10.5	1322	End stage Lewy body disease	12
**PD57**	90	F	14	1134	Bronchopneumonia	NA
**PD60**	80	M	18	1336	Urosepsis	NA
**PD63**	91	F	5	1119.6	Parkinson’s disease	22
**PD65**	67	M	2.25	1033.1	Parkinson’s disease	9
**PD66**	73	M	17.5	1376.4	Aspiration pneumonia	22
**PD67**	65	M	17	1223.7	Pneumonia	12
**PD69**	84	F	22	1139	deconditioning, PD	NA
**PD71**	80	M	5.5	1143	Pneumonia	9
**PD73**	83	F	4	1120	Pneumonia/PD	NA
**PD77**	76	F	6.5	1074.3	Abdominal carcinoma	23
**PD78**	80	M	5.5	NA	Parkinson’s disease	NA
**PD79**	77	M	6.5	1279.5	End stage Lewy body disease	22
**PD105**	82	M	20	1364.9	Lewy body dementia	NA

### Formalin-fixed paraffin-embedded tissue processing and tissue microarray construction

Upon receipt of the brain, the right hemisphere of each brain was fixed by perfusion of 15% formaldehyde in 0.1 M phosphate buffer through the cerebral arteries and subsequently dissected into anatomically significant blocks. 5 mm-thick cuts were sampled from each block for paraffin embedding. The brain tissue blocks were processed for paraffin embedding as previously described [10, 11]. A tissue microarray (TMA) was constructed using 2 mm paraffin-embedded formalin-fixed cores of PD (n = 22) and neurologically normal controls (n = 22) middle temporal gyrus (MTG) grey matter, as described previously [12]. The MTG was selected due to its relatively homogeneous distribution of α-Synuclein (α-Syn) pathology and involvement during later disease stages. All paraffin blocks were sequentially sectioned using a rotary microtome (Leica Biosystems, RM2335) at a thickness of 7 μm. Sections were individually mounted onto Über plus charged microscope slides (IntstrumeC) using a 41°C-water bath (Leica Biosystems, H1210).

### Fluorescent immunohistochemistry

7 μm-thick sections from paraffin-embedded MTG blocks or tissue microarrays (TMA) were fluorescently stained as previously described [13–16]. The detailed step-by-step standard protocol (10 mM tris-EDTA pH 9 heated to 121°C in a pressure cooker) can be found on protocols.io (DOI: dx.doi.org/10.17504/protocols.io.5qpvo3wdzv4o/v1). The primary antibodies used in this study (four SMARCC2/Baf170 antibodies: Mouse SMARCC2, PCRP-SMARCC2-1A3, Developmental Studies Hybridoma Bank, 1:100; Rabbit SMARCC2, Origene (AP06744U-N), 1:100; Rabbit Baf170, Abcam (ab71907),1:300; Ms Baf170, Santa Cruz Biotechnology (sc17838), 1:100; Rabbit α-synuclein-phospho S129, ab51253, Abcam, 1:3000; Guinea Pig Neun, ABN90, Millipore, 1:500; Guinea Pig p62, GP52-c, Progen, 1:500, Rat pTFP-43, BL829901, clone ID3, BioLegend, 1:3000). These antibodies have been extensively validated and used previously [10, 17]. Control sections where the primary antibody was omitted showed no immunoreactivity. The control experiments showed that the secondary antibodies displayed no cross-reactivity.

### Cyclic Heat-Induced Epitope Retrieval (CHIER)

Epitope retrieval is paramount for the detection of proteins using immunohistochemistry. Cyclic Heat-Induced Epitope Retrieval (CHIER) is a novel cyclic heating technique developed in-house that enhances the detection of SMARCC2 compared to other standard antigen retrieval protocols. Four CHIER variations were included in the analysis. These variations include changes in temperature, incubation times and number of cycles. The antigen retrieval protocols were performed on sequential MTG sections. A detailed step-by-step CHIER protocol can be found on protocols.io (DOI: dx.doi.org/10.17504/protocols.io.kxygx3284g8j/v1).

### Image acquisition and quantification

Whole-tissue images were acquired using an automated fluorescence microscope (Zeiss Axioimager Z2) equipped with a MetaSystems VSlide slide scanner (MetaSystems) and Colibri 7 light source, running Metafer 5 (v4.4.114) with a Plan-Apochromat 20x/0.8 NA dry objective lens. Images were stitched using the MetaCyte software. Sections stained for SMARCC2 were used for the quantification as described previously [13]. The images were extracted from VSViewer, and the rips and fold were segmented out from each section on FIJI/ImageJ (V 2.14.0/1.54f) using the polygon selection tool. Following the segmentation, a precise measurement of the area was made.

To obtain a background staining intensity measure for the SMARCC2 staining, a 50 μm × 50 μm square (area = 2500 μm^2^) was placed over three different areas of background staining, and the grayscale pixel value was measured. The background measurements were averaged to give a mean background staining intensity value. The multipoint tool was used to determine the number of cells with SMARCC2^+^ cytobodies. To be counted, a cell needed a SMARCC2^+^ cytobody in a cell as delineated by NeuN labelling and be located close to the nucleus. If this criterion was satisfied, the single largest aggregate close to the nucleus was selected with the multipoint tool. Once all the apparent cytobodies were selected, the grayscale pixel value was measured for every selection point. To be considered a true aggregate, the grayscale pixel value for each selection point had to be higher than a predetermined threshold (45 grayscale points). To obtain total SMARCC2^+^ cytobody cell density values (cells/mm^2^), the total number of SMARCC2^+^ cytobody cells counted for each case was then divided by the measured area. For tissue microarray core analysis, approximately 4mm^2^ for each case was quantified. For the treatment comparison on MTG sections, approximately 4–4.5 mm^2^ for each case was quantified. Two individuals (BF and BVD) counted while blinded to the case number and disease status.

The diameters of cytobodies were measured using the straight line tool in ImageJ (NIH, Bethesda, MD). Measurements were performed on calibrated images. For each cytobody, the straight line tool was used to draw a line across the widest part of the cytobody, defining this as the diameter. Care was taken to position the line from one edge of the cytobody to the opposite edge without overshooting the boundary. Measurements were repeated across 120 cytobody samples from neurologically normal (n: 5) and PD cases (n: 5).

Super-resolution images were acquired using an LSM 800 with an Airyscan confocal microscope (Zeiss) with a 63x/1.4 NA Plan Apochromat DIC M27 oil immersion objective lens and GaAsp-PMT detector. Images were acquired using the built-in Airyscan module and processed using the ZEN microscopy software (Zeiss). All images were acquired using optimal Nyquist sampling parameters, and those acquired in a Z-series used the optimal step size of 0.13 μm. Stimulated emission depletion (STED) images were acquired using an Abberior Facility STED microscope (60x UPLXAPO oil immersion lens, 1.42 NA) using ImSpector Lightbox software (Specim, v.16.3.13779). A 561-nm pulsed diode laser was used to excite Alexa Fluor 594. A pulsed 775-nm laser was used for STED imaging to deplete both fluorophores. The Dapi channel was not depleted. After scanning, the images were processed using the PureDenoise plugin for ImageJ (National Institutes of Health, USA v1.53f51). All deconvolution of STED images and 3D reconstruction of aggregates was performed using the Huygens Professional software package (Scientific Volume Imaging, Hilversum, The Netherlands) [4].

### Western blot

Protein for western blot analysis was extracted using sample harvesting buffer (62.5 mM Tris-HCl pH 6.8, 2% SDS, 10% glycerol), subsequently denatured in NuPage LDS sample buffer (ThermoFisher Scientific; NP0007) at 85°C for 5 min and loaded into 4–12% Bis-Tris gels (ThermoFisher Scientific; NP0336BOX) to be resolved via SDS-PAGE, using a MOPS SDS running buffer (ThermoFisher Scientific; J62847-AP) with a dual colour Protein Ladder (BioRad; 1610374). Gels were transferred with NuPage transfer buffer (ThermoFisher Scientific; NP0006) onto methanol-activated PVDF membranes (Millipore, IPFL00005) for 1 h using a constant voltage of 20 V. Membranes were blocked for 1 h at room temperature in a 1:1 solution of Intercept® (TBS) Blocking Buffer (LI-COR, 927–60001) and TBS-T (TBS with 0.01% Tween 20). Primary antibodies (Mouse SMARCC2, PCRP-SMARCC2-1A3, Developmental Studies Hybridoma Bank, 1:100; Rabbit SMARCC2, Origene (AP06744U-N), 1:2500; Rabbit Baf170, Abcam (ab71907),1:300) were incubated overnight at 4°C in a blocking buffer. Membranes were washed in TBS-T (3 × 10 min) and incubated with secondary antibodies (diluted 1:10,000 in blocking buffer with 0.02% SDS) for 3 h at room temperature, protected from light. Membranes were washed in TBS-T (3 × 10 min), in TBS (10 min) and imaged using a BioRad ChemiDocTM MP Imaging System [4] (Full uncropped Western blots: S1 Raw image).

### Statistical analysis

Data visualisation and statistical hypothesis testing were performed using GraphPad Prism® Version 9.00. Normality testing was performed using the D’Agostino-Pearson normality test. Mann-Whitney test was used to compare groups. Correlations were determined using Spearman correlation. Statistical significance was set at *p* < 0.05. Final figure were composed using Adobe Photoshop CC (Adobe Systems Incorporated, v23.1). Statistical significance was set as *p* < 0.05.

## Results

### SMARCC2 localisation in the middle temporal gyrus (MTG) grey matter

**Fig 1:**
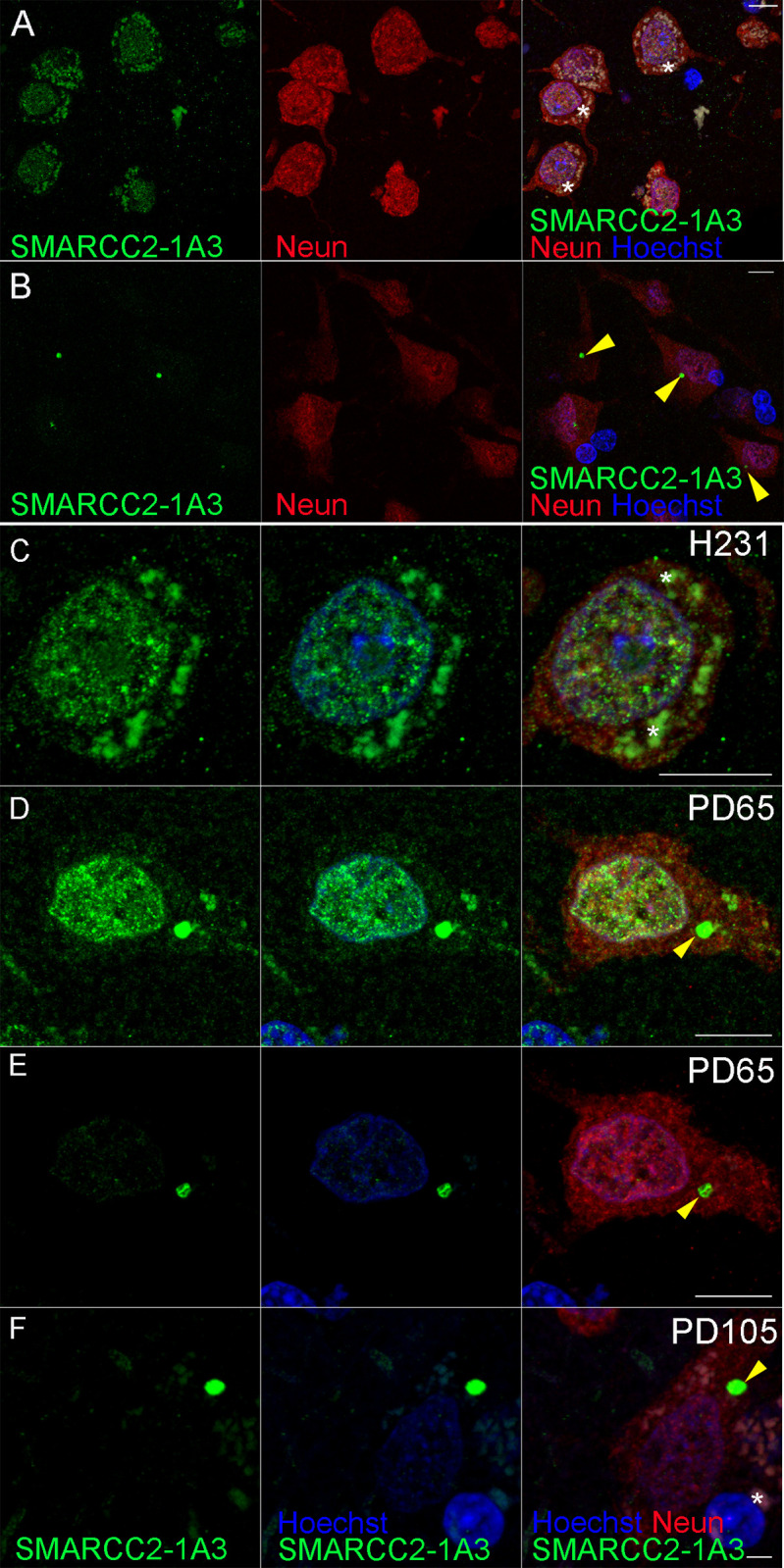
SMARCC2-1A3 labelling in the middle temporal gyrus brain tissue (MTG). SMARCC2-1A3 (green), NeuN (red), nucleus (blue). (A) In controls, we primarily observed a nuclear punctate SMARCC2 staining pattern. (B) In some controls and all PD cases, circular cytoplasmic SMARCC2^+^ bodies, named SMARCC2^+^ cytobodies, are present. (C) Typical nuclear SMARCC2^+^ labelling. (D) Nuclear SMARCC2^+^ labelling with a SMARCC2^+^ cytobody. (E) The same cell as D was imaged using the optimal setting for SMARCC2^+^ cytobody visualisation. (F) No nuclear SMARCC2^+^ labelling with saturated SMARCC2^+^ cytobody (same image setting as in E). All staining shown in Fig 1 was performed using standard antigen retrieval protocol (10 mM tris-EDTA pH 9). Scale bars represent 10 μm.

We observed SMARCC2-1A3 labelling in the nucleus using our standard antigen retrieval protocol. This SMARCC2-1A3 labelling was punctate and present within NeuN^+^ neurons. Spotty staining was observed in the cytoplasm (A). As expected, non-specific lipofuscin autofluorescence was also present in these neurons (Fig 1A, indicated by *). In a subset of MTG neurons, intense SMARCC2-1A3^+^ labelling was observed in the cytoplasm. SMARCC2-1A3 labelling was present as a circular cytoplasmic body in these cells, which we named cytobodies. These SMARCC2^+^ cytobodies were found in neurologically normal and PD cases (Fig 1B). If present, then most commonly, only one SMARCC2^+^ cytobody/cell was found. However, some cells contained between 1–4 SMARCC2^+^ cytobodies/cell. SMARCC2^+^ cytobodies were only found in neurons. Overall, the cellular SMARCC2-1A3 staining based on observations from all neurologically normal and PD cases could be categorised as (1) Nuclear punctate SMARCC2-1A3, (2) nuclear punctate SMARCC2-1A3 with a SMARCC2^+^ cytobody, (3) weak to no nuclear SMARCC2-1A3 with a SMARCC2^+^ cytobody or (4) no SMARCC2-1A3 labelling (Fig 1A–1F). The staining intensity of the SMARCC2^+^ cytobodies was higher than the nuclear punctate labelling. This large intensity difference resulted in an apparent lack of nuclear SMARCC2-1A3 labelling in some cells despite SMARCC2-1A3 labelling still being present, as demonstrated (Fig 1D and 1E). Here, a typical neuron with nuclear punctate SMARCC2-1A3 labelling and a SMARCC2^+^ cytobody is imaged using two different integration times. Fig 1D is imaged using the parameters (normal integration) identical to Fig 1C, whereas Fig 1E imaging (short exposure) is optimised to avoid saturation of the SMARCC2^+^ cytobody. This short exposure still saturated SMARCC2^+^ cytobodies in other cases (e.g. PD105), where only neurons with SMARCC2^+^ cytobodies that lack nuclear SMARCC2-1A3 labelling were found (Fig 1F).

**Fig 2:**
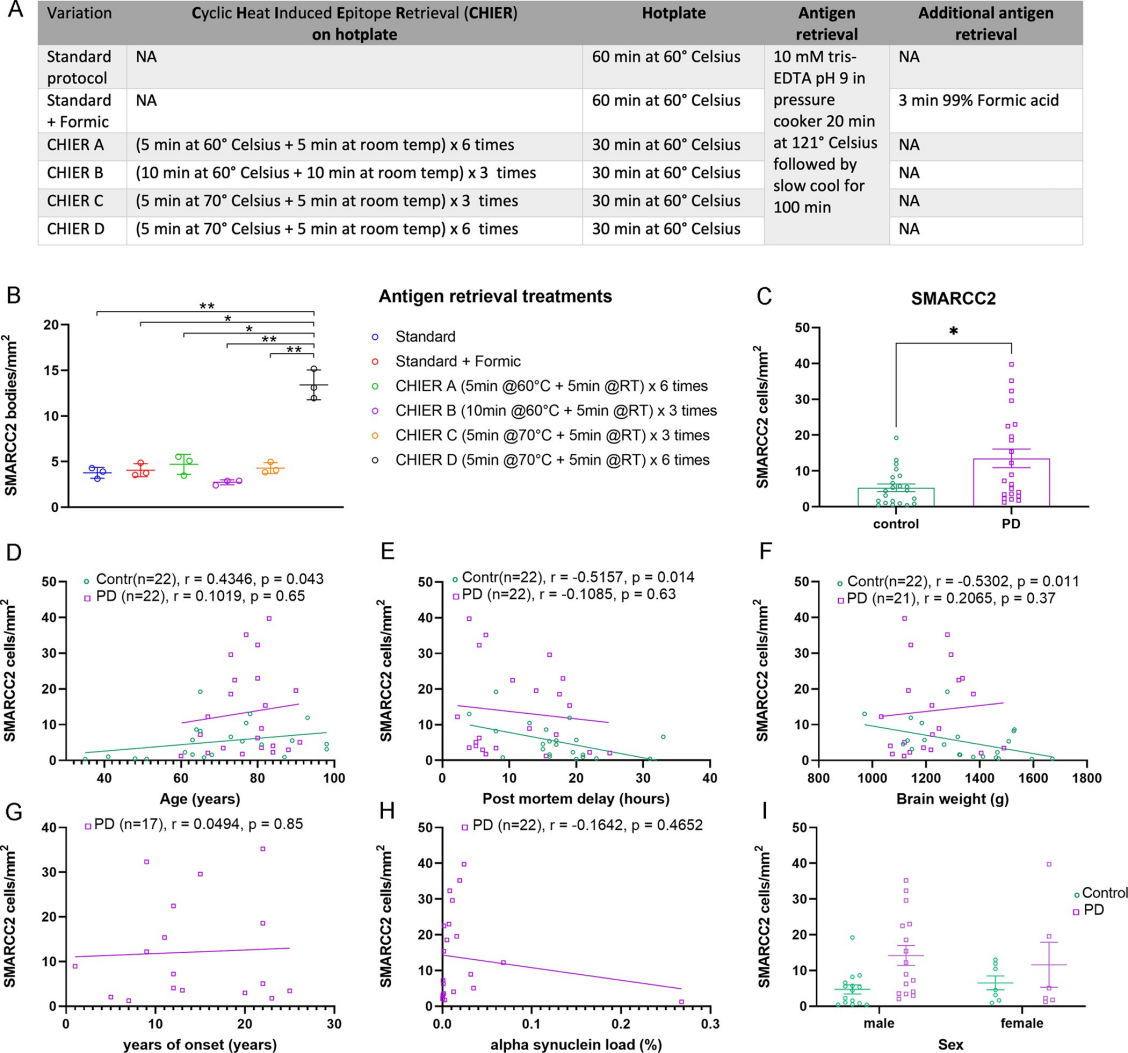
Quantification of neurons with SMARCC2+ cytobodies using different Antigen retrieval methods. (A) Antigen retrieval optimisation methods. (B) Comparison Cyclic Heat Induced Epitope Retrieval (CHIER) to standard and formic acid antigen retrieval using PD cases. (C) Quantification of neurons with SMARCC2^+^ cytobodies in MTG tissue (neurologically normal and PD). Correlation of neurons with SMARCC2^+^ cytobodies with (D) age of death, (E) post-mortem delay, (F) brain weight, (G) years of onset, (H) α-Syn pS129 load. (I) Cell count with SMARCC2^+^ cytobodies separated male/female. (C-I) Staining performed with CHIER protocol D. * p <0.05, ** p <0.01.

As part of this research, we tested additional antigen retrieval steps to our standard Tris-EDTA protocol to improve SMARCC2 labelling (Fig 2A) [10,18–20]. Cyclic Heat-Induced Epitope Retrieval (CHIER) is a novel cyclic heating technique developed in-house using a sequence of heating and cooling tissue slides on a hot plate. We compared different CHIER variations to standard and standard + formic using tissue from PD cases. In the cases with high numbers of neurons with SMARCC2^+^ cytobodies, all antigen retrieval variations, including the standard antigen retrieval protocol, detected cytobodies. Acid treatment, commonly used to increase the labelling of aggregated proteins, did not improve SMARCC2^+^ cytobodies detection. The number of neurons with SMARCC2^+^ cytobodies was quantified for each variation. CHIER variation D ((5 min at 70° Celsius + 5 min at room temp) repeated six times) improved the SMARCC^+^ staining significantly compared to all other antigen retrievals. CHIER D (13.4 ± 1.63) resulted in a three-fold increase in the number of neurons with SMARCC2^+^ cytobodies/mm^2^ compared to the standard antigen retrieval (60 min hotplate at 60° Celsius with tris EDTA ph9 with Tween in the pressure cooker) (3.77 ± 0.60; Fig 1 in S1 Appendix, Fig 2B).

We used our optimised antigen retrieval method (CHIER D) to quantify the neurons with SMARCC2^+^ cytobodies in MTG grey matter. We found an increase of neurons with SMARCC2^+^ cytobodies/mm^2^ in PD (13.5 ± 12.2 in PD vs 5.3 ± 5.0 in neurologically normal cases, p = 0.014) (Fig 2C). We subsequently correlated our counts with age at death, post-mortem delay, brain weight, years of PD onset and α-Syn load. No strong correlations or differences between sexes were found. Moderate correlations were present between neurons with SMARCC2^+^ cytobodies and age at death (r = 0.4346; p = 0.043), post-mortem delay (r = -0.5157; p = 0.014) and brain weight (r = -0.5302; p = 0.011) (Fig 2D–2I).

**Fig 3:**
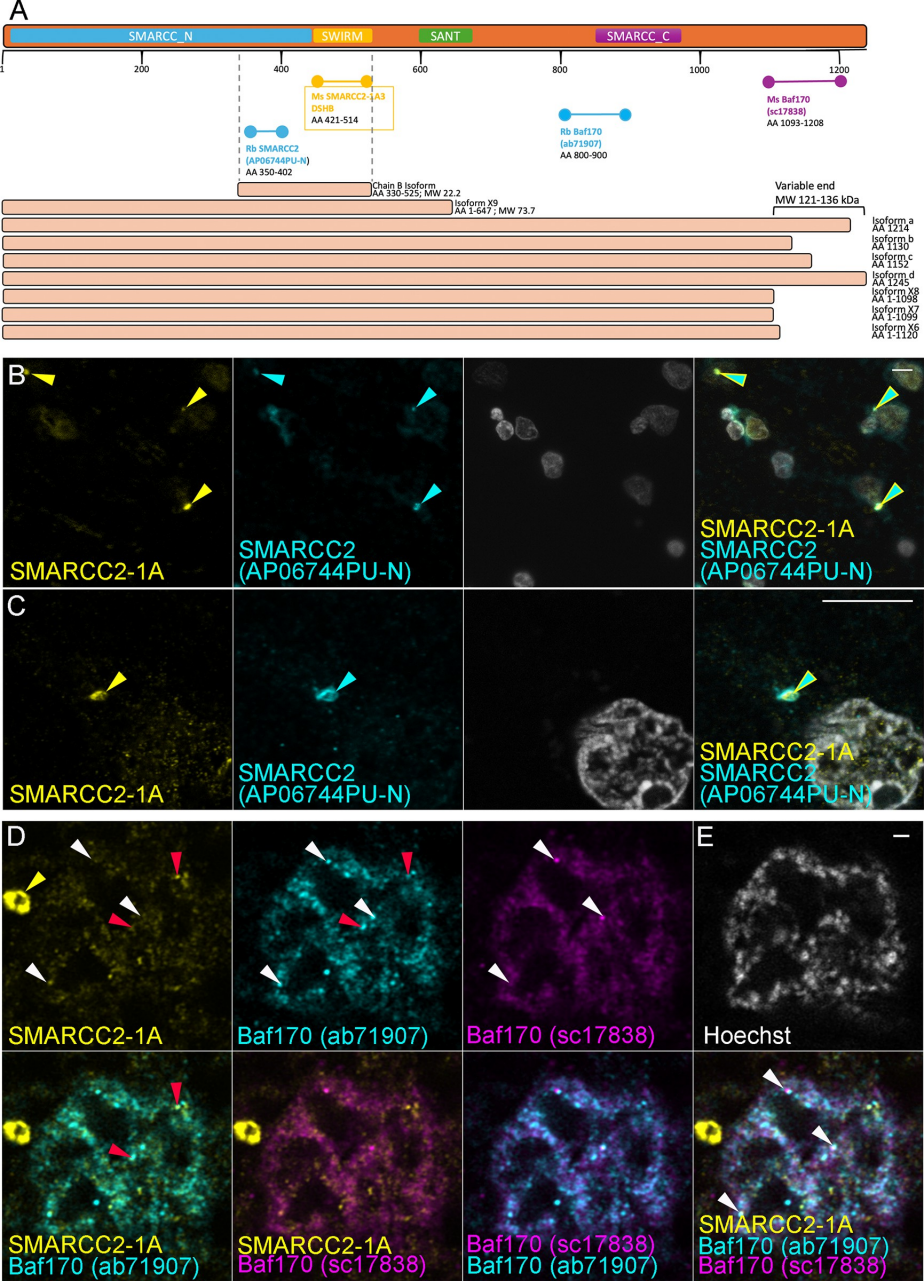
SMARCC2/Baf170 antibody comparison. (A) Schematic diagram depicting the amino acid sequence and structural domains of the SMARCC2 protein, including the various isoforms and an antibody-epitope map of the four epitope-specific SMARCC2/Baf170 antibodies used in this study. (B-C) SMARCC-1A3 (yellow) and SMARCC2 (APO6744PU-N, cyan) labels SMARCC2^+^ cytobodies (yellow and cyan arrows) and nuclear puncta (weak labelling for SMARCC2 (APO6744PU-N). (D) SMARCC2 (ab71907, cyan) and Baf170 (sc17838, magenta) only label nuclear puncta, of which some overlap (red and white arrows) with SMARCC2-1A3 puncta (yellow). SMARCC2 (ab71907, cyan) and Baf170 (sc17838, magenta) never label SMARCC2^+^ cytobodies as seen for SMARCC2-1A3 and SMARCC2 (APO6744PU-N). All labelling in this Fig was performed using CHIER protocol D. Scale bars represent 10 μm for B & C and 1 μm for D.

Up to now, only nuclear SMARCC2 labelling has been described. Therefore, to cross-validate SMARCC2-1A3 labelling of SMARCC2^+^ cytobodies, we tested three additional SMARCC2/Baf170 antibodies targeting the central and C-terminal ends of SMARCC2 (Fig 3A). SMARCC2 (APO06744PU-N) labelled nuclear puncta and cytobodies similarly to SMARCC2-1A3 (Fig 3B and 3C, yellow and cyan arrows). SMARCC2 (ab71907) and Baf170 (sc17838) never labelled SMARCC2^+^ cytobodies as observed for SMARCC2-1A3 and SMARCC2 (APO06744PU-N) and only labelled nuclear puncta, some of which overlapped with SMARCC2-1A3 (white and red arrows; Fig 3B and 3D).

**Fig 4:**
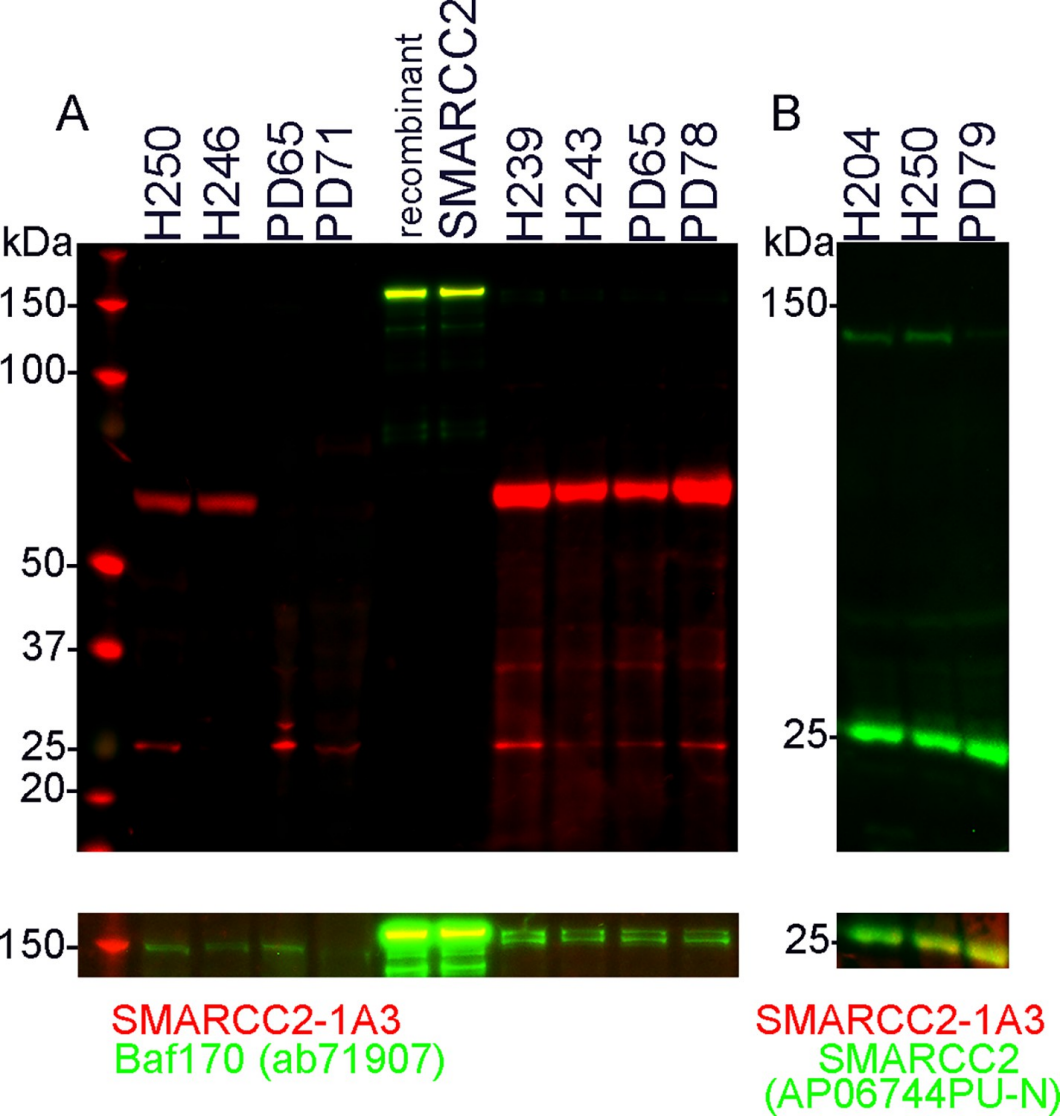
Western blot of human middle temporal gyrus (MTG) brain tissue, pericytes and recombinant human SMARCC2 protein. (A) Western blotting of the positive control containing full-length SMARCC2 protein (TP303774, Origene) with SMARCC2-1A3 (red) showed a clear band at 150 kDa, and a faint band at 130 kDa, which overlaps with the SMARCC2 (ab71907; green) bands observed at 150 and 130 kDa. A high-exposure image of the 150 kDa region is shown underneath the entire blot. In MTG (H250, H246, PD65, PD71) and pericytes (H239, H243, PD65, PD78), two clear bands at 60 and 25 kDa were observed with SMARCC2-1A3 and a light band at 150 kDa with SMARCC2 (ab71907). (B) Western blotting of MTG with SMARCC2 (AP06744PU-N, green) detected two clear bands at 130 kDa and 25 kDa, with the 25 kDa band overlapping with the 25 kDa SMARCC2-1A3 band (red). A high-exposure image of the 25 kDa region is shown underneath the entire blot.

To investigate if truncated SMARCC2 and isoforms are present, protein extracts of the middle temporal gyrus (MTG) and primary pericyte cultures from MTG were analysed. Western blotting of the positive control containing full-length SMARCC2 protein (TP303774, Origene) with SMARCC2-1A3 showed a clear band at 150 kDa as expected and a faint band at 130 kDa. These bands overlap with the SMARCC2 (ab71907) bands observed at 150 and 130 kDa (High exposure image of 150 kDa region shown underneath entire blot; Fig 4A). Two clear bands at 60 kDa and 25 kDa were observed in the MTG extracts with SMARCC2-1A3. In PD cases, the 60 kDa SMARCC2-1A3 band was less intense to absent, whereas in control cases, the 25 kDa SMARCC2-1A3 band was not consistently observed. SMARCC2 (ab71907) only detected a 150 kDa band in the MTG tissue (Fig 4A). Western blotting with SMARCC2 (AP06744PU-N) detected two clear bands at 130 kDa and 25 kDa, with the 25 kDa band overlapping with the 25 kDa SMARCC2-1A3 band (High exposure image of 25 kDa region shown underneath entire blot; Fig 4B).

### 3D architecture SMARCC2^+^ cytobodies

**Fig 5:**
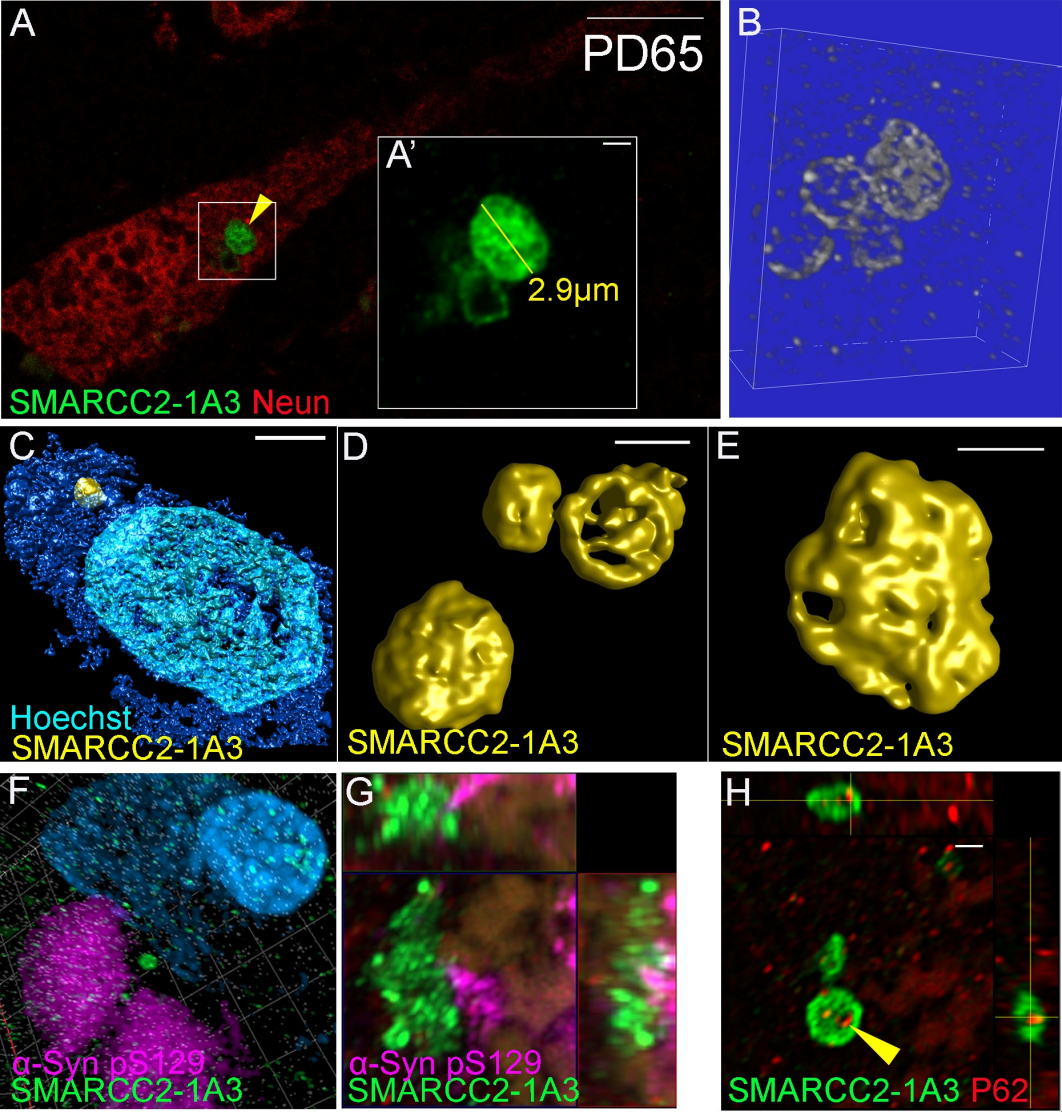
Super-resolution imaging of neurons with SMARCC2^+^ cytobodies. (A) single confocal plane image of neurons with SMARCC2^+^ cytobody. (A’) zoom of SMARCC2^+^ cytobody (B) 3D STED render of SMARCC2^+^ cytobody shown in A. (C) 3D render of neuron with SMARCC2^+^ cytobody. (D-E) 3D renders of SMARCC2^+^ cytobodies. (F) SMARCC2^+^ cytobodies not interacting with α-Syn pS129 aggregates. (G) SMARCC2^+^ cytobodies interacting with α-Syn pS129 aggregates (single confocal plane with orthogonal views). (H) P62 is found within SMARCC2^+^ cytobodies (single confocal plane with orthogonal views). Labelling performed using optimised CHIER protocol D. Scale bars represent 10 μm for A & C and 1 μm for A’, D-H.

CHIER is compatible with advanced fluorescent techniques with super-resolution imaging of the SMARCC2^+^ cytobodies, revealing a more complex structure resembling a honeycomb architecture. The staining observed in the SMARCC2^+^ cytobodies resembles a spherical structure with an intricate, lattice-like pattern akin to the internal framework of a cellular matrix. The average diameter of the SMARCC2^+^ cytobodies was 1.8 μm, ranging between 1 and 3.8 μm. Even though most SMARCC2^+^ cytobodies were single circular structures, some were more complex and consisted of multiple fused cytobodies (Fig 5A–5E, S1, S2 Videos)

Due to the similarities with aggregate pathology observed in other neurodegenerative diseases, we investigated potential interactions between SMARCC2^+^ cytobodies and α-Syn and p62. CHIER did not negatively affect the labelling staining of these antibodies. Overall, SMARCC2^+^ cytobodies did not co-localise with α-Syn pathology (labelled by pS129 α-Syn; Fig 5F), although a rare example did make contact with an α-Syn aggregate (Fig 5G). Furthermore, occasionally, p62 was found within SMARCC2^+^ cytobodies (Fig 5).

## Discussion

Our study enhanced the well-established Tris-EDTA antigen retrieval protocol by incorporating additional antigen retrieval steps. Notably, formic acid treatment, a technique often employed to improve aggregated protein detection, had little effect. Cyclic Heat-Induced Epitope Retrieval (CHIER), a novel technique we developed, increased the detection of SMARCC2^+^ cytobodies threefold. This method involves heating and cooling the tissue slides on a hot plate. CHIER variation D, which involves a cycle of 5 minutes at 70° Celsius followed by 5 minutes at room temperature, repeated six times, proved the most effective. In our hands, CHIER had no deleterious effects on other antibody detections (Neun, α-Syn pS129, pTDP43, Fig 2 in S1 Appendix) or Ulex Eurapaeus Lectin (endothelial marker, Fig 2 in S1 Appendix), nor did it negatively affect detection with super-resolution microscopy. This novel variation expands the repertoire of antigen retrieval options with CHIER, combining the antigen retrieval benefits of HIER and formic acid treatment.

Emerging studies indicate dysfunction in chromatin remodelling processes in neurodegenerative disorders, including PD [9]. α-Synuclein (overexpression disturbs the SWI/SNF complex [21]. α-Syn is a key player in PD, and the abnormal accumulation of α-Syn aggregates is a pathological hallmark of PD (reviewed in [22]). However, the direct mechanism by which this occurs remains unknown. Our study found limited evidence of direct interaction between SMARCC2^+^ cytobodies and α-Syn (p-S129) labelled aggregates.

To our knowledge, the presence of SMARCC2^+^ cytobodies in neurons or any other cell type has not been studied. By applying CHIER, we could accurately quantify the amount of SMARCC2^+^ cytobodies in MTG. By doing so, we observed nuclear and cytoplasmic SMARCC2 with a significant increase of neurons with SMARCC2^+^ cytobodies in PD. The staining observed in the SMARCC2^+^ cytobodies resembles a complex spherical structure with an intricate, lattice-like pattern akin to the internal framework of a cellular matrix. This distinctive configuration resembling honeycomb candy suggests a complex and organised arrangement indicative of specific biological processes or structural functionalities in the cellular environment. The lattice-like pattern of the SMARCC2^+^ antibodies implies that other proteins are likely interacting with SMARCC2. The presence of p62 in some of the SMARCC2^+^ cytobodies indicates that these cytobodies are partially destined to be degraded by autophagy. In this aspect, SMARCC2 resembles the translocation from the nucleus to the cytoplasm seen for TDP-43 protein. In ALS, TDP-43 undergoes cytoplasmic mislocalisation and aggregation, along with a myriad of post-translational modifications such as phosphorylation, ubiquitination, and truncation, altering its structure and function [23]. Although this comparison is purely speculative, as this manuscript did not investigate phosphorylation or ubiquitination, it does warrant further investigation into why SMARCC2^+^ cytobodies are increased in PD.

Previous studies identified the crucial and multifaceted role of SMARCC2 in SWI/SNF complexes. Even though SWI/SNF complexes contain the same core proteins (SMARCC1, SMARCC2, SMARCD1), numerous variable subunits provide each complex with a distinct identity that modulates its function [7]. It is likely that through this mechanism of variable subunits in SWI/SNF complexes, SMARCC2-mediated gene regulation is altered [24]. Combined with the known SMARCC2 isoforms and/or SMARCC2 truncational variants, this could explain why only a partial overlap was observed for the nuclear puncta detected with the four SMARC2/Baf170 antibodies (Fig 3B–3D).

The C-terminus SMARCC2/Baf170 antibodies did not label the SMARCC2 cytobodies, whereas the antibodies detecting the central part of the proteins did. This differential immunolabeling combined with the identification of lower molecular weight SMARCC2 bands (60 and 25kDa; Fig 4) suggests that the SMARCC2^+^ cytobodies found in neurons contain a short isoform or a truncated SMARCC2, being 25 or 60 kDa in size. It is important to highlight that SMARCC2-1A3 targets the SWIRM domain, which interacts directly with DNA, mediating specific protein-protein interactions to assemble chromatin-protein complexes [25]. Our data does not preclude the presence of truncated SMARCC2 from the nucleus, which still contains the SWIRM domain, but does add to the complex role of SMARCC2 in chromatin regulation and neurodegenerative diseases such as PD.

## Conclusions

Cyclic Heat-Induced Epitope Retrieval (CHIER) is a new antigen retrieval protocol that can significantly improve the detection of hard-to-detect epitopes. Using CHIER, we observed a notable increase in SMARCC2-positive cytoplasmic bodies within neurons of the MTG in PD, a phenomenon not previously reported in PD research. This novel finding warrants further investigation to elucidate the origins, associated interacting proteins, and potential implications of truncated SMARCC2 accumulation in these cytoplasmic bodies to better understand their role in PD. Additionally, CHIER does not compromise the antigenicity of other antibodies, supporting its broader application in multiplex fluorescent immunohistochemistry and super-resolution imaging, with the potential for improving the detection of other chromatin-binding or aggregated proteins.

## Supporting information

S1 Appendix Fluorescent labelling standard antigen retrieval compared to CHIER. (PDF)

S1 Raw image Uncropped Western blots. (PDF)

S1 Video 3D render of SMARCC2^+^ cytobody in neuron shown in Fig 3 A. Labelling in this video was performed using CHIER protocol D.
(AVI)

S2 Video 3D render of SMARCC2^+^ cytobody in neuron shown in Fig 3 C. SMARCC2+ (yellow), NeuN (cyan), Hoechst (blue). Labelling in this video was performed using CHIER protocol D.
(AVI)

S1 File (PDF)
