# Supplementary material for: Enhanced detection of distinct honeycomb-structured neuronal SMARCC2 cytobodies in Parkinson’s Disease via Cyclic Heat-Induced Epitope Retrieval (CHIER)
Source: PLoS One. 2024 Dec 17;19(12):e0315183. doi: 10.1371/journal.pone.0315183 (PMC11651576; doi:10.1371/journal.pone.0315183)
Supplement: S1 Appendix — (PDF) [file pone.0315183.s001.pdf]

Variation	Cyclic Heat Induced Epitope Retrieval (CHIER) on hotplate	Hotplate	Antigen retrieval	Additional antigen retrieval
Standard protocol	NA	60 min at 60° Celsius	10 mM tris-EDTA pH 9 in pressure cooker 20 min at 121° Celsius followed by slow cool for 100 min	NA
Standard + Formic	NA	60 min at 60° Celsius		3 min 99% Formic acid
CHIER A	(5 min at 60° Celsius + 5 min at room temp) x 6 times	30 min at 60° Celsius		NA
CHIER B	(10 min at 60° Celsius + 10 min at room temp) x 3 times	30 min at 60° Celsius		NA
CHIER C	(5 min at 70° Celsius + 5 min at room temp) x 3 times	30 min at 60° Celsius		NA
CHIER D	(5 min at 70° Celsius + 5 min at room temp) x 6 times	30 min at 60° Celsius		NA

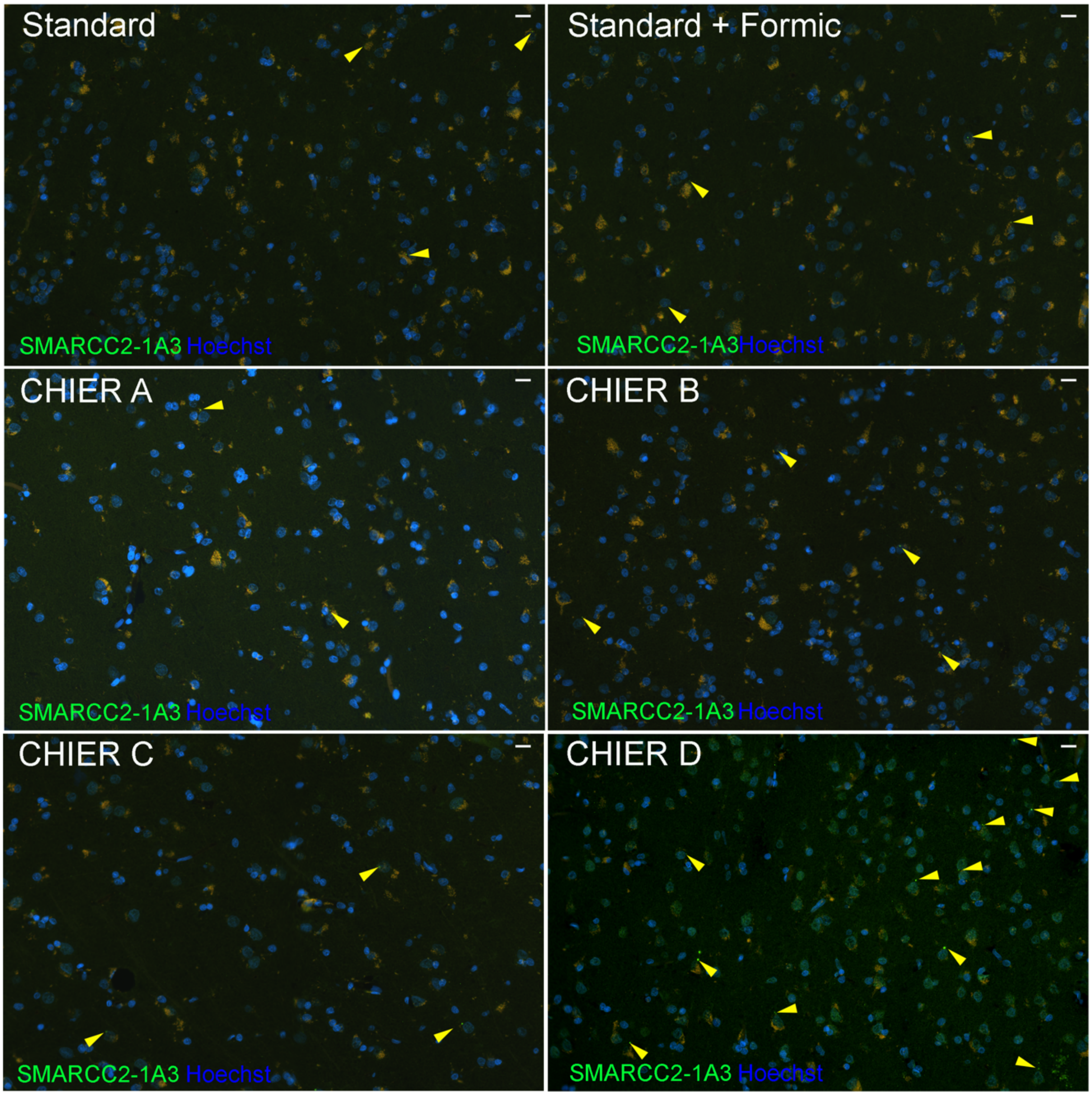

Appendix Figure 1: Antigen retrieval protocol and immunofluorescent labelling of SMARCC2 using standard, standard + formic acid or Cyclic Heat Induced Epitope Retrieval (CHIER) variations. Yellow arrows indicate cells with SMARCC2^{+} cytobody. Scale bars represent 20 µm.

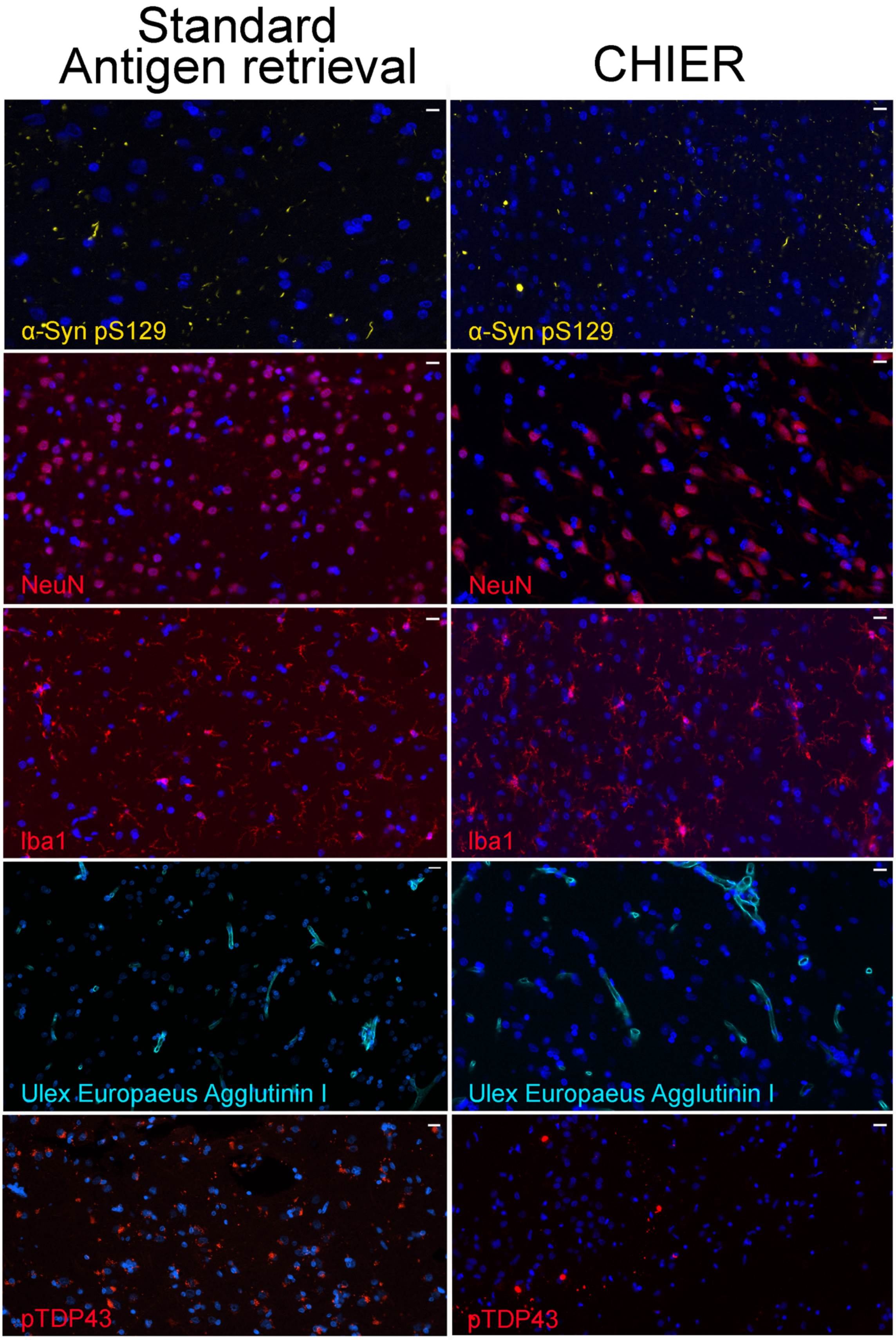

Appendix Figure 2 Immunofluorescent labelling of α-Syn S129, NeuN, Iba1, pTDP43 Ulex Europaeus Agglutinin 1 using standard or Cyclic Heat Induced Epitope Retrieval (CHIER DU). Scale bars represent 20 μm.
