# Supplementary figures and images for: Enhanced detection of distinct honeycomb-structured neuronal SMARCC2 cytobodies in Parkinson’s Disease via Cyclic Heat-Induced Epitope Retrieval (CHIER)
Source: PLoS One. 2024 Dec 17;19(12):e0315183. doi: 10.1371/journal.pone.0315183 (PMC11651576; doi:10.1371/journal.pone.0315183)

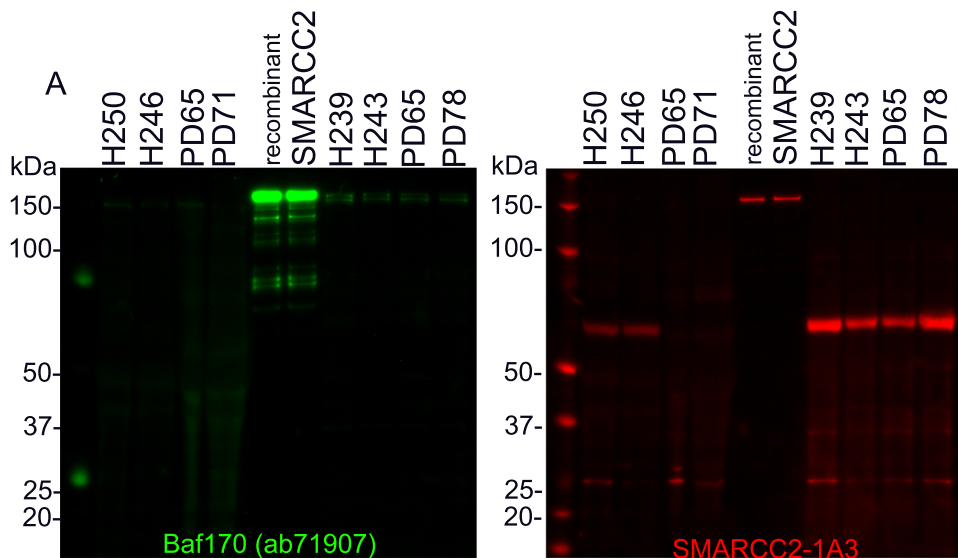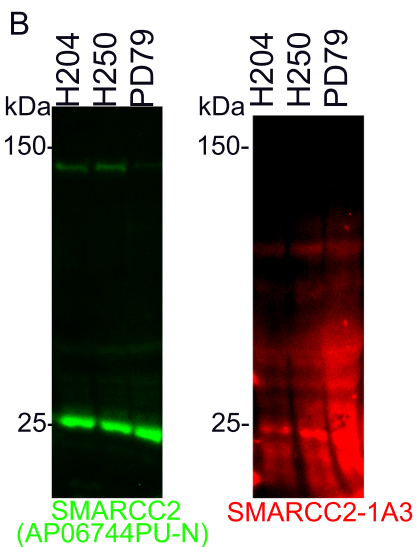

Supplement: S1 Raw image — (PDF) [file pone.0315183.s002.pdf]
