# Supplementary material for: Enhanced detection of distinct honeycomb-structured neuronal SMARCC2 cytobodies in Parkinson’s Disease via Cyclic Heat-Induced Epitope Retrieval (CHIER)
Source: PLoS One. 2024 Dec 17;19(12):e0315183. doi: 10.1371/journal.pone.0315183 (PMC11651576; doi:10.1371/journal.pone.0315183)
Supplement: S1 File — (PDF) [file pone.0315183.s005.pdf]

Nov 26, 2024

Immunofluorescent labelling of paraffin brain tissue sections

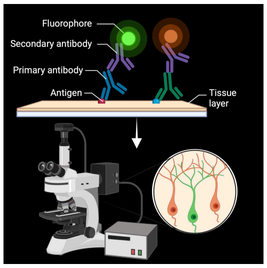

DOI
dx.doi.org/10.17504/protocols.io.5qpvo3wdzv4o/v1

Birger Victor Dieriks[1,2], Helen Murray[1,2]

[1]Department Anatomy and Medical Imaging, The University of Auckland;
[2]Centre for Brain Research, The University of Auckland

Birger Victor Dieriks
The University of Auckland

OPEN ACCESS

DOI: dx.doi.org/10.17504/protocols.io.5qpvo3wdzv4o/v1

Protocol Citation: Birger Victor Dieriks, Helen Murray 2024. Immunofluorescent labelling of paraffin brain tissue sections. **protocols.io** **https://dx.doi.org/10.17504/protocols.io.5qpvo3wdzv4o/v1**

License: This is an open access protocol distributed under the terms of the **Creative Commons Attribution License**, which permits unrestricted use, distribution, and reproduction in any medium, provided the original author and source are credited

Protocol status: Working
We use this protocol and it's working

Created: December 08, 2023

Last Modified: November 26, 2024

Protocol Integer ID: 91988

Keywords: Paraffin Immunohistochemistry, IHC labelling protocol, immunofluorescence protocol, labelling brain sections, immunohistochemistry

Funders Acknowledgement:

Health Research Council Hercus Fellowship
Grant ID: 21/034

Royal Society of New Zealand Te Apārangi - Catalyst Seeding
Grant ID: 22-UOA-049-CSG

Neurological Foundation of New Zealand
Grant ID: 2026 PRG

Health Ed Trust
Grant ID: Postdoctoral Fellowship

Health Research Council of New Zealand Emerging Researcher First Grant
Grant ID: 21/646

Health Research Council of New Zealand Programme Grant
Grant ID: 21/710

Abstract

This immunofluorescence protocol permits the labelling of multiple antibodies on one paraffin tissue section. This protocol details the potential variations that detect the protein of interest using fluorescently labelled secondary antibodies.

Immunohistochemistry (IHC) combines anatomical, immunological and biochemical techniques to image discrete tissue components by using appropriately labelled antibodies to bind specifically to their target antigens in situ. IHC makes it possible to visualize and document the high-resolution distribution and localization of specific cellular components within cells and their proper histological context. There are multiple approaches and permutations in IHC methodology.

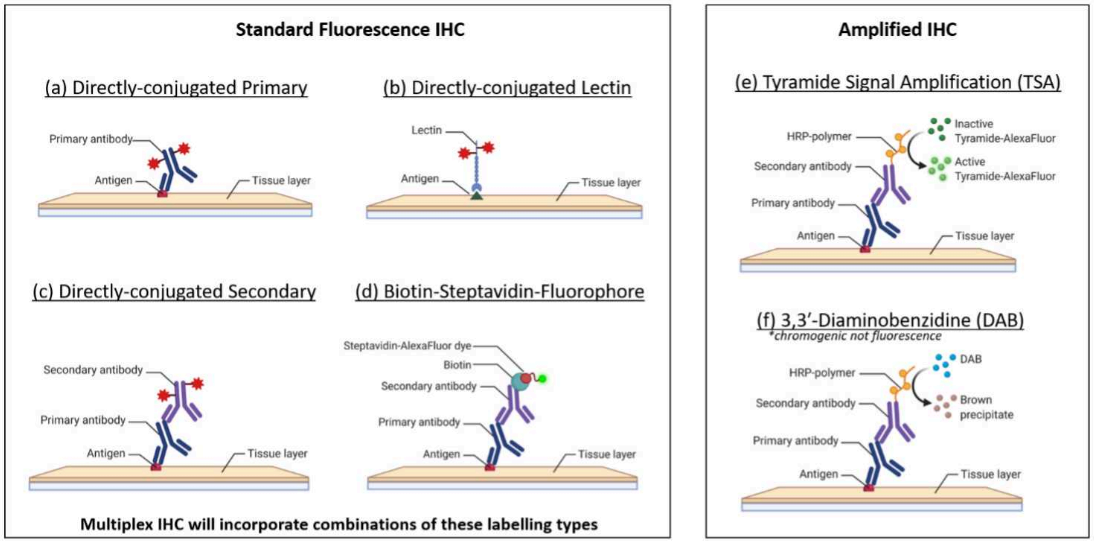

Figure 1. Different approaches for IHC (image by Dr Helen Murray and Dr Victor Dieriks)

Immunofluorescence permits labelling of multiple antibodies on one tissue section. For double or triple labelling, the primary antibodies must be raised in different species e.g. mouse, rabbit, chicken, guinea pig or goat. The corresponding secondary antibodies are conjugated to fluorophores with non-overlapping excitation/emission spectra (commonly used are fluorophores that excite at 488, 594 and 647nm). Antibodies from the same species but different Ig subtypes (e.g. mouse IgG1, IgG2a, IgG2b, IgG3, IgM) can be combined only if secondary antibodies that are highly specific to these subtypes are used. The specific excitation and emission wavelengths of different fluorophores permits labelling of multiple antibodies on one tissue section and visualisation of the labelling using widefield fluorescence or confocal microscopy. Hoechst or DAPI are commonly used nuclear counterstains for immunofluorescence.

Amplified IHC methods include fluorescent tyramide signal amplification (TSA, Figure 1e) and chromogenic 3,3'-Diaminobenzidine (DAB, Figure 1f) labelling. These methods detect the primary antibody using a secondary antibody conjugated to the horse-radish peroxidase (HRP) enzyme (Figure 2a). Alternatively, a biotin-conjugated secondary antibody can be applied, followed by a streptavidin conjugated HRP (Figure 2b). For chromogenic labelling, HRP converts the soluble substrate DAB into an insoluble brown product that can be visualised using brightfield microscopy. For fluorescence labelling, HRP converts an inactive tyramide-fluorophore complex into an active fluorescent complex that can be visualized using fluorescence microscopy.

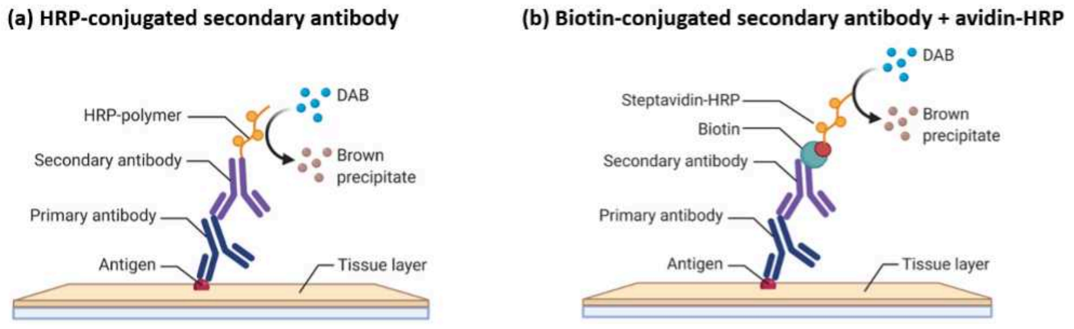

Figure 2. Different permutations of amplified IHC (image by Dr Helen Murray and Dr Victor Dieriks)

Counterstains Nissl is a common nuclear counterstain for chromogenic labelling. Hoechst or DAPI are common fluorescent dyes used to stain DNA for fluorescent counterstaining.

Guidelines

Heat block reaches 60° Celcius and can cause burns when skin comes into contact with the hot surface.

Materials

Statmark Permanent marking pen - alcohol and solvent-resistant (Statlab)
Antigen retrieval buffers
Sodium citrate buffer ([M] 10 millimolar (mM) Sodium citrate, [M] 0.05 % (v/v) Tween 20, pH 6.0)

- Tri-sodium citrate (dihydrate) 2.94 g.
- Distilled water 1 L.
- Mix to dissolve. Adjust to pH 6 with 1N HCl.
- Add 0.5 mL Tween 20 and mix well. Store at room temperature for three months or at 4 °C for more extended storage.

Tris-EDTA buffer ([M] 10 millimolar (mM) Tris base, 1 mM EDTA solution, [M] 0.05 % (v/v) Tween 20, pH 9.0)

- Tris 1.21 g.
- EDTA 0.37 g.
- Distilled water 1 L.
- Mix to dissolve. Adjust pH to pH 9 .
- Add 0.5 mL Tween 20 and mix well. Store at room temperature for three months or at 4 °C for more extended storage.

Buffers can be used without detergent if your antibody labelling is sensitive to detergent.
Sodium citrate buffer ([M] 10 millimolar (mM) Sodium citrate, [M] 0.05 % (v/v) **without** Tween 20, pH 6.0)

- Tri-sodium citrate (dihydrate) 2.94 g.
- Distilled water 1 L.
- Mix to dissolve. Adjust to pH 6 with 1N HCl.
- Store at room temperature for three months or at 4 °C for more extended storage.

Tris-EDTA buffer ([M] 10 millimolar (mM) Tris base, 1 mM EDTA solution, [M] 0.05 % (v/v) **without** Tween 20, pH 9.0)

- Tris 1.21 g.
- EDTA 0.37 g.
- Distilled water 1 L.
- Mix to dissolve. Adjust pH to pH 9 .
- Store at room temperature for three months or at 4 °C for more extended storage.

ImmEdge Pen (Vector Laboratories, cat H-4000)
TrueBlack (Biotum, cat 23007)
dH20
PBS
NGS
NDS
triton-x100
tween 20
Hoechst

an intermediate dilution for Hoechst can be made and stored for up to one year. Dilute Hoechst 1:500 in H2O. DO NOT USE PBS for this step, as Hoechst is unstable in phosphate buffers.

add full antibody list here MAYBE INCLUDE COMMONLY USED PRIMARIES

A	B	C	D	E	F	G
**Antibody**	**Target**	**Species**	**Isotype**	**Manufacturer**	**Catalogue No.**	**Dilution**
Goat anti-mouse AlexaFluor 488	Mouse	Goat	IgG (H+L)	ThermoFisher	A11001	1:500
Goat anti-mouse AlexaFluor 488	Mouse	Goat	IgG1	ThermoFisher	A21121	1:500
Goat anti-mouse AlexaFluor 488	Mouse	Goat	IgG2a	ThermoFisher	A21131	1:500
Goat anti-rabbit AlexaFluor 488	Rabbit	Goat	IgG (H+L)	ThermoFisher	A11034	1:500
Goat anti-chicken AlexaFluor 488	Chicken	Goat	IgG (H+L)	ThermoFisher	A11039	1:500
Goat anti-mouse AlexaFluor 594	Mouse	Goat	IgG (H+L)	ThermoFisher	A11032	1:500
Goat anti-mouse AlexaFluor 594	Mouse	Goat	IgG1	ThermoFisher	A21125	1:500
Goat anti-mouse AlexaFluor 594	Mouse	Goat	IgG2a	ThermoFisher	A21135	1:500
Goat anti-rabbit AlexaFluor 594	Rabbit	Goat	IgG (H+L)	ThermoFisher	A11037	1:500
Goat anti-chicken AlexaFluor 594	Chicken	Goat	IgG (H+L)	ThermoFisher	A21449	1:500
Goat anti-mouse AlexaFluor 647	Mouse	Goat	IgG2c	Jackson ImmunoResearch	115-607-188	1:500
Goat anti-guinea pig AlexaFluor 647	Guinea Pig	Goat	IgG (H+L)	ThermoFisher	A21450	1:500
Goat anti-rabbit AlexaFluor 647	Rabbit	Goat	IgG (H+L)	ThermoFisher	A21245	1:500
Goat anti-chicken AlexaFluor 647	Chicken	Goat	IgG (H+L)	ThermoFisher	A21449	1:500
Goat anti-rabbit AlexaFluor 800	Rabbit	Goat	IgG (H+L)	LI-COR	926-32211	1:500
Donkey anti-goat AlexaFluor 647	Goat	Donkey	IgG (H+L)	ThermoFisher	A21447	1:500

Safety warnings

Heat block reaches 60° Celcius and can cause burns when skin comes into contact with the hot surface.
When using acids work in the fume cabinet avoiding spills and skin contact.
Make sure chemical waste is disposed of according to on-site regulations.

Ethics statement

The human post-mortem brain tissue used in this project was sourced from the Neurological Foundation Human Brain Bank (Centre for Brain Research, University of Auckland, New Zealand). All brain tissue was donated upon written, informed consent from the individual(s) and/or next of kin before brain removal.
Please ensure consent is obtained, and all protocols required used are approved by the relevant Ethics Committee.

Slide preparation

1 Label slides with a Statmark pen or a pencil.

Note

Any other ink will wash off during dewaxing in step 3

Always include the proper controls for any fluorescent labelling. Control sections where the primary antibody was omitted should show no immunoreactivity.

2 Formalin-fixed paraffin-embedded tissue sections, when mounted on glass slides for microscopic examination, are placed on a hotplate with the tissue side up. This practice serves to

- Facilitate Tissue Adherence: The hotplate softens the paraffin, allowing the tissue to flatten and firmly adhere to the glass slide. This ensures the tissue's integrity and correct orientation during staining and microscopic analysis.
- Effectively Dehydrate: Paraffin sections often retain residual water. The hotplate aids in the removal of this water by evaporation, ensuring proper dehydration. Adequate dehydration is critical for subsequent staining and the preservation of tissue structures.

2.1 - Place all slides on a hot plate (Leica HI1220) at 60 °C for up to 01:00:00 . 1h

Note

This step cooks the Statmark ink to the slide and melts the wax. 01:00:00 is the maximum; 20 minutes is sufficient if you are pushed for time.

Dewaxing 1h 20m

3 Immediately following hotplate incubation, sections were submerged in 1h 5m

00:30:00 2 x 100% xylene baths to remove paraffin wax.

Subsequently, tissue sections were rehydrated by submerging slides in a series of alcohol baths

00:15:00 2 x 100% EtOH

00:05:00 95% EtOH

00:05:00 80% EtOH

00:05:00 75% EtOH

00:05:00 3 x distilled H_2O (dH_2O) to wash off any remaining EtOH.

Antigen retrieval in pressure cooker

1h 40m

4 Fill the pressure Cooker with approx. 600ml dH2O.

4.1 Add slides in the cassette holder. Add the antigen retrieval buffer to the pressure cooker cartridge (Biovendor). Make sure to add sufficient antigen retrieval buffer to cover the tissue, but not to overfill

Frequently used buffers:

- Sodium citrate buffer pH6
- Tris-EDTA buffer pH9

> **Note**
>
> The antigen retrieval buffer depends on the antibodies you are using. This needs to be optimised by testing the various options.

4.2 Press start. 2h

This will initiate a heating protocol where the pressure cooker reaches 121 °C at 1 bar for 00:20:00 , followed by an 01:40:00 of gradual cool down.

> **Note**
>
> Cassettes with slides can be left in the pressure cooker after 20 min heating or taken out and placed at room temperature. This will not affect the staining.

4.3 Pour out the antigen retrieval solution and replace it with 1x dH_2O. Repeat 2x for about 00:05:00 per wash. 5m

Use the pressure cooker cartridges for the wash steps in the rest of the protocol.

4.4 go to step #7 if no additional antigen retrieval is required.

OPTIONAL Formic Acid Antigen retrieval

5 Embedding tissue in paraffin often leads to the masking of antigenic sites due to the formation of cross-links between proteins, which can hinder the binding of antibodies to their target

antigens. Formic acid, a strong acid, reverses this masking effect. It works by breaking down these protein cross-links, thus exposing the antigenic sites that were previously hidden. This additional formic acid antigen retrieval is especially beneficial for detecting certain types of antigens, such as prion proteins and other aggregated proteins, such as alpha-synuclein and Tau.

5.1 Tap dry slides and add 99% formic acid for 00:04:00 at Room temperature 4m

Note

Formic acid is a strong acid and should be used and dispersed in a fume cabinet following the local Health and Safety requirements.

5.2 Wash immediately with dH20 3x 00:05:00 per wash. 5m

5.3 go to step #7 if no additional antigen retrieval is required.

OPTIONAL Hydrochloric Acid Antigen retrieval

6 Hydrochloric Acid (HCl) antigen retrieval is particularly effective for visualizing nuclear antigens in paraffin-embedded tissue sections. By breaking down the protein cross-links that mask these antigens during formalin fixation, HCl enhances the exposure and binding of antibodies to nuclear epitopes, thus improving the clarity and specificity of immunohistochemical staining.

CITATION

Coppieters N, Dieriks BV, Lill C, Faull RL, Curtis MA, Dragunow M (2014). Global changes in DNA methylation and hydroxymethylation in Alzheimer's disease human brain..

LINK

https://doi.org/10.1016/j.neurobiolaging.2013.11.031

6.1 Tap dry slides and add 2N HCL for 00:05:00 at Room temperature . 5m

Note

Hydrochloric Acid is a strong acid and should be used and dispersed in a fume cabinet following the local Health and Safety requirements.

6.2 Wash immediately with dH20 3x 00:05:00 per wash. 5m

6.3 go to step #10 if no additional antigen retrieval is required.

Wax borders

7 Draw a hydrophobic barrier around each tissue section to reduce the reagents required.
This step can be done during the 3 x dH_2O washes

Note

The wax borders must be drawn before permeabilisation because the wax will not stick to the slide if it is coated in detergent.

7.1 Drain dH2O from the slides one slide at a time, but DO NOT let sections dry out. Draw a hydrophobic barrier around each tissue section using the ImmEdge pen (Vector Laboratories). To conserve reagents, draw barriers close to, but not on, sections. Tissue with a wax pen on top has high autofluorescence.
Place the slide back into dH2O until all slides are done.

Note

The ImmEdge pen draws a hydrophobic barrier around each tissue section, but sometimes an excess amount of liquid flows out of the pen. To avoid this, always test the pen on tissue paper when starting a new exp.
The wax may not bind well to some brands of glass slide if there is residual dH2O on the glass. Use a paper towel to dry the area of glass carefully where you want to draw the wax border, but be careful not to touch the tissue.
Exposing the wax border to water before it has adequately dried will result in it being compromised

Permeablisation

8 Once wax borders are completed, replace dH2O with PBS-T and incubate for 00:15:00 at 4 °C . 15m

Note

This step can be omitted if your antibody labelling is sensitive to detergent. This should be optimised for each antibody.

8.1 Wash slides 3x 00:05:00 in 1x PBS. 5m

8.2 go to step #10 if TrueBlack treatment is not required.

OPTIONAL TrueBlack autofluorescence quencher.

9 Trueblack treatment reduces the autofluorescence that is present in human brain sections. However, this additional step is only required when the signal-to-noise ratio of your labelling is low. TrueBlack can improve the signal for low-intensity labeling by reducing the background.

9.1 Wash sections 3x 00:05:00 in dH2O. PBS will cause the TrueBlack to clump on the tissue. 5m

9.2 Dilute TrueBlack (Biotum) reagent 1:20 in 70% EtOH (make 50µl per section depending on tissue size). Make sufficient true black solution to adequately cover the tissue section

9.3 Place sections on a tray and add TrueBlack solution to sections for 00:00:30 30s

Note

When adding a small amount of TrueBlack, a portion of the section can dry out. Therefore, we recommend placing the slides on a tray and swirling the slides gently to evenly distribute the Trueblack.

9.4 Wash sections 3x 00:05:00 in dH2O. PBS will cause the TrueBlack to clump on the tissue. 5m

9.5 Wash sections 3x 00:05:00 in PBS. 5m

Blocking for non specific labelling

10 In this step, we will block potential non-specific labelling. Ideally, this is performed using serum from animals in which the secondary antibodies were made (eg. if using goat secondary antibodies, then use goat serum; if using donkey secondary antibodies, then donkey serum). In practice, both can be used. HOWEVER, when a primary goat antibody is used, one cannot use goat serum in this protocol, as the secondary anti-goat antibody would label the entire section as it is covered with goat serum.

10.1 Make a humidified slide chamber by adding wet paper towels to one of the plastic slide chambers or trays. You can also use this time to check if you need to make a 10% blocking buffer and defrost the serum from -20 °C freezer if needed.

10.2 Make a 10% serum-blocking solution

- 500 µl serum stock solution + 4.5 ml 1x PBS. Use goat or donkey serum, depending on the species of your secondary antibodies. 10% serum solutions should be stored at 4 °C and discarded after two weeks. 10% serum solutions can be stored at -20 °C and repeatedly freeze/thawed.

10.3 Drain excess PBS from slides and move to the humid chamber. Add 100-200 µl of 10% blocking solution to each slide. Ensure the tissue is completely covered to the wax border. The volume of serum needed depends on the size of the tissue and doesn't have to be precise. Tissue needs to be entirely covered.

Primary antibody incubation

11 For immunolabeling techniques like immunofluorescence, selecting the appropriate antibody is pivotal for the specificity and sensitivity of the assay. Antibodies serve as tools to specifically detect target antigens within a sample, and the precision of this detection largely hinges on the antibody's quality and specificity. The source of the antibody, marked by the supplier's reliability and reputation, plays a significant role since antibodies can differ substantially in their quality, purity, and overall performance based on their origin. Consequently, it is essential for researchers to meticulously assess their experimental needs and choose antibodies that align optimally with the requirements of their specific applications. This careful selection is key to ensuring the immunolabeling results are accurate.

11.1 Make immunobuffer: 1% normal goat (NGS) or donkey serum (NDS) in PBS

- 1 ml 10% NGS (or NDS) from step 6 + 9 ml 1x PBS. 1% serum solutions should be stored at 4 °C and discarded after two weeks. 1% serum solutions can be stored at -20 °C and repeatedly freeze/thawed.

11.2 Dilute antibodies in immunobuffer.
Dilution factors depend on the company, individual antibody and region of interest.

Note

For double or triple labelling, the primary antibodies must be raised in different species, e.g., mice, rabbits, chickens, guinea pigs, or goats. The corresponding secondary antibodies are conjugated to fluorophores with non-overlapping excitation/emission spectra (commonly used are fluorophores that excite at 488, 594 and 647nm). Antibodies from the same species but different Ig subtypes (e.g. mouse IgG1, IgG2a, IgG2b, IgG3, IgM) can be combined only if highly specific secondary antibodies to these subtypes are used.

Thaw/store antibodies on ice during the protocol. Once thawed vortex and centrifuge before taking the required amount.

11.3 Remove the 10% serum solution from the sections and return the slides to the humid chamber. No wash step is required here because the primary antibodies are diluted in the same serum.

11.4 Add 100-200 µl of antibody solution to each slide. Volume can be optimised in the blocking step.

Note

The entire section must be covered with the antibody solution, as any dried-out sections will have increased autofluorescence.

11.5 Incubate at 4 °C overnight.

Note

As we use small volumes, sections can dry out during the overnight incubation. It is critical there is an excess amount of liquid in the humid chamber and it is well sealed.

Washing off primary antibody dilution

5m

12 Antibody solution can be removed and discarded or recovered for additional antibody incubations. In our hands, we have successfully re-used primary antibody solutions up to three times. Store at -4 °C for up to two weeks or at -20 °C for long-term storage.

12.1 Remove the primary antibody solution and transfer the slides back to the pressure cooker cartridge for 3 x 00:05:00 washes with 1x PBS. 5m

12.2 go to step #10 if peroxidase blocking is not required.

VARIATION** Block endogenous peroxidases. 15m

13 This step is required for DAB labelling, Tyramide Signal Amplification (TSA) or any labelling that involves peroxidase-based signal amplification.

13.1 Make methanol blocking solution: 50% methanol + 1% H2O2 in MilliQ water
Incubate at Room temperature for 00:10:00 — 10m

13.2 Wash 3x 00:05:00 with 1x PBS and continue to the secondary antibody incubation step — 5m

Secondary antibody incubation 15m

14 Secondary antibodies can be incubated — 3h

- overnight at 4 °C or
- at Room temperature for 03:00:00

14.1 Dilute secondary antibodies 1:500 in immunobuffer. Add Hoechst diluted at 1:20,000 If doing fluorescence labelling. Hoechst counterstaining does not interact with secondary binding, so it is more efficient to include it in this step.

> **Note**
>
> A 1:500 dilution is used for secondary antibodies, such as ThermoFisher, Jackson ImmunoResearch, and Santa Cruz. This dilution works well and we have found that changing secondary antibody dilution has minimal impact on the overall staining.

14.2 Transfer slides to the humid chamber and add 100-200µl of secondary antibody solution to each slide

14.3 Incubate at Room temperature for 03:00:00 . Cover the slide chamber with tinfoil to protect fluorophores from light. — 3h

14.4 Remove the secondary antibody solution.
Transfer the slides back to the pressure cooker cartridge for 3 x 00:05:00 washes with 1x PBS and continue to coverslipping — 5m

VARIATION** Tyramide amplification 15m

15 We use Alexa Flour 488/594/647 Tyramide Superboost Kits (ThermoFisher) for primary antibody signal amplification. If additional secondary antibodies are simultaneously required, — 3h 5m

then these were diluted in 1x poly-HRP conjugated goat anti-mouse IgG secondary antibody (B40912-B, ThermoFisher) and added to the sections for 03:00:00 at Room temperature as described above.

Note

Poly-HRP conjugated goat anti-mouse IgG secondary antibody incubation can occur simultaneously with fluorescently conjugated secondaries from go to step #14.1

Following incubation, the secondary antibody solutions were aspirated from slides and underwent 3 x 00:05:00 PBS washes.

15.1 Prepare Tyramide working solution by diluting one drop of 100x hydrogen peroxide (H2O2) (B40912-
C2, ThermoFisher) in 1mL of MilliQ water for a 1:20 dilution.
Dilute one drop of 20x Reaction buffer (B40912-C3, Invitrogen) in 1mL of MilliQ water.
Add the diluted 100x hydrogen peroxide solution to the diluted 20x reaction buffer at 1:100 dilution.

15.2 Heat the combined solution 37 °C for 00:05:00 5m
Add Alex Flour 488 Tyramide Reagent (B40912 Invitrogen) to the warm reaction buffer at a 1:100 dilution to produce a final Tyramide working solution.
Make this solution fresh and use it immediately.

15.3 Add 100-200 µl of the Tyramide working solution to each slide and incubate at 37 °C for 00:10:00 10m

15.4 Wash 3x 00:05:00 with 1x PBS and continue to coverslipping. 5m

Coverslipping

16 Coverslip sections with Prolong gold.

Note

Different antifade reagents are available. Human brain sections labelled with up to six fluorophores and coverslipped ProLong Gold (ThermoFisher) antifade provide good protection against oxidation and bleaching.

16.1 Add a small amount of ProLong Gold (ThermoFisher) to the coverslip.
Invert the glass slide so the tissue is between the coverslip and the glass slide.

Gently push the glass slide on the coverslip, avoiding air bubbles and allowing the ProLong Gold to spread over the entire tissue section.

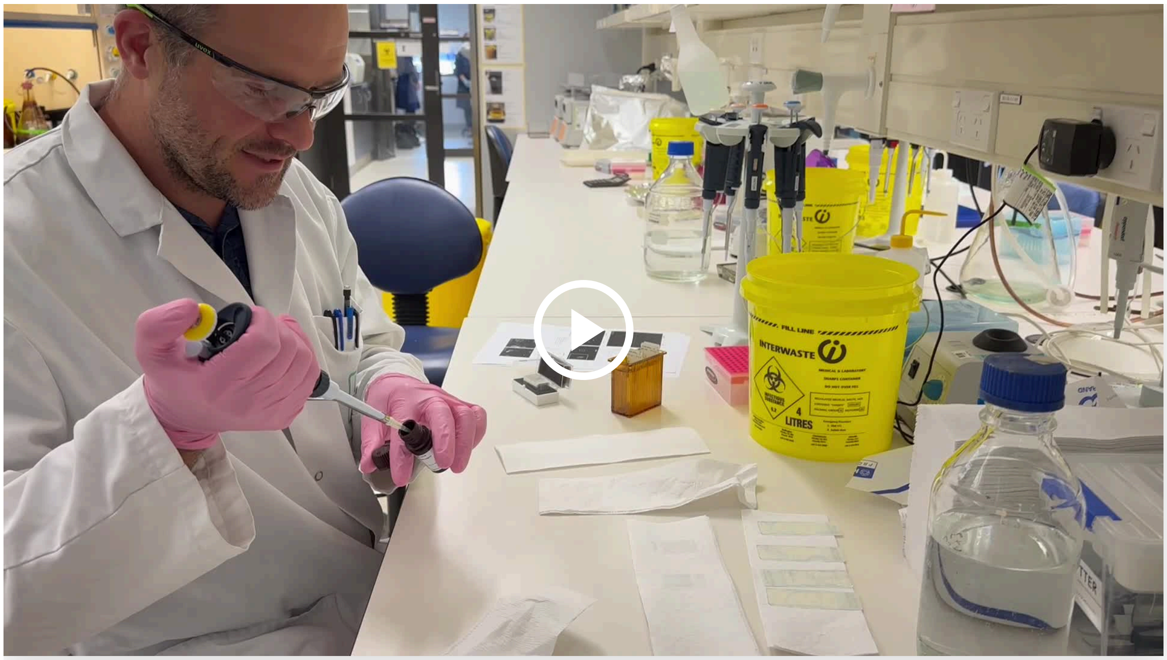

16.2 An alternative method for coverslipping:
Gently push the coverslip on the glass slide, avoiding air bubbles and allowing the ProLong Gold to spread over the entire tissue section.

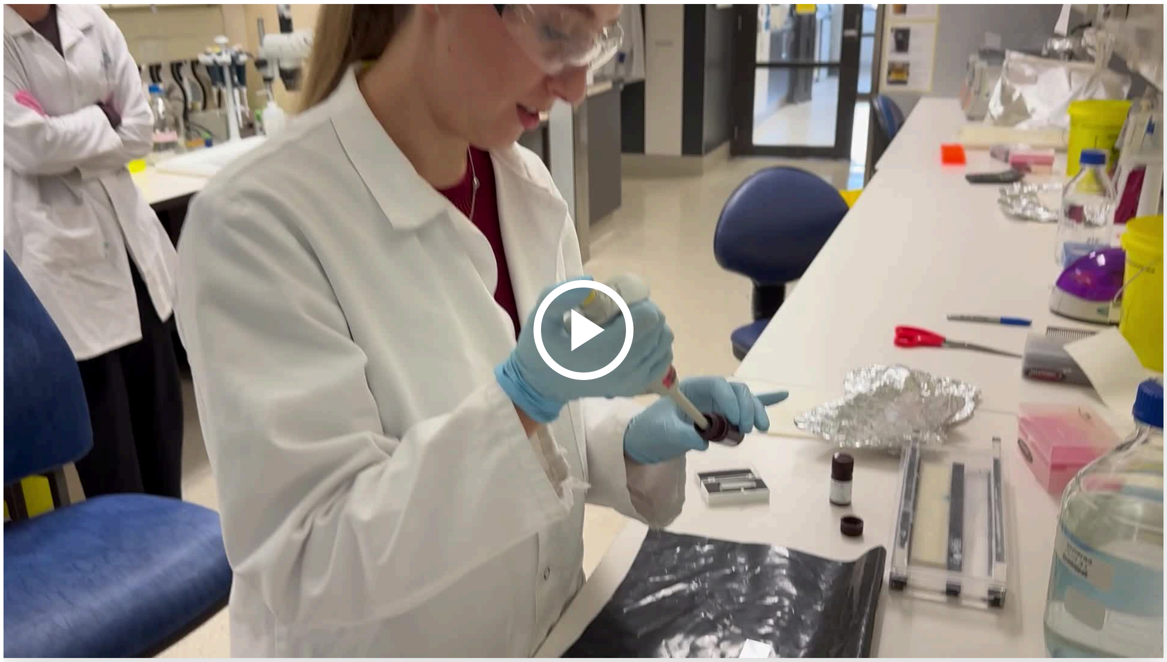

16.3 Edges of cautofluorescenceoverslips can be sealed with nail polish.

Note

Avoid nail polishing over the tissue, as the nail polish generates autofluorescence.

Storage

17 Store sections at 4 °C in slide boxes protected from light.

Note

Ideally, imaging of tissue sections should be performed as soon as possible for optimal results. However, in our hands, labelling remains present for up to five years if stored at optimal conditions.

Protocol references

The protocol detailed here was used in the following studies:

CITATION

Dieriks BV, Park TI, Fourie C, Faull RL, Dragunow M, Curtis MA (2017). α-synuclein transfer through tunneling nanotubes occurs in SH-SY5Y cells and primary brain pericytes from Parkinson's disease patients..

LINK

https://doi.org/10.1038/srep42984

CITATION

Stevenson TJ, Murray HC, Turner C, Faull RLM, Dieriks BV, Curtis MA (2020). α-synuclein inclusions are abundant in non-neuronal cells in the anterior olfactory nucleus of the Parkinson's disease olfactory bulb..

LINK

https://doi.org/10.1038/s41598-020-63412-x

CITATION

Murray HC, Low VF, Swanson ME, Dieriks BV, Turner C, Faull RL, Curtis MA (2016). Distribution of PSA-NCAM in normal, Alzheimer's and Parkinson's disease human brain..

LINK

https://doi.org/10.1016/j.neuroscience.2016.06.003

CITATION

Highet B, Dieriks BV, Murray HC, Faull RLM, Curtis MA (2020). Huntingtin Aggregates in the Olfactory Bulb in Huntington's Disease..

LINK

https://doi.org/10.3389/fnagi.2020.00261

CITATION

Murray HC, Dieriks BV, Swanson MEV, Anekal PV, Turner C, Faull RLM, Belluscio L, Koretsky A, Curtis MA (2020). The unfolded protein response is activated in the olfactory system in Alzheimer's disease..

LINK

https://doi.org/10.1186/s40478-020-00986-7

CITATION

Murray HC, Swanson MEV, Dieriks BV, Turner C, Faull RLM, Curtis MA (2018). Neurochemical Characterization of PSA-NCAM(+) Cells in the Human Brain and Phenotypic Quantification in Alzheimer's Disease Entorhinal Cortex..

LINK

https://doi.org/10.1016/j.neuroscience.2017.12.019

CITATION

Dieriks BV, Dean JM, Aronica E, Waldvogel HJ, Faull RLM, Curtis MA (2018). Differential Fatty Acid-Binding Protein Expression in Persistent Radial Glia in the Human and Sheep Subventricular Zone..

LINK

https://doi.org/10.1159/000487633

CITATION

Jansson D, Dieriks VB, Rustenhoven J, Smyth LCD, Scotter E, Aalderink M, Feng S, Johnson R, Schweder P, Mee E, Heppner P, Turner C, Curtis M, Faull R, Dragunow M (2021). Cardiac glycosides target barrier inflammation of the vasculature, meninges and choroid plexus..

LINK

https://doi.org/10.1038/s42003-021-01787-x

CITATION

Gardner B, Dieriks BV, Cameron S, Mendis LHS, Turner C, Faull RLM, Curtis MA (2017). Metal concentrations and distributions in the human olfactory bulb in Parkinson's disease..

LINK

https://doi.org/10.1038/s41598-017-10659-6

CITATION

Coppieters N, Dieriks BV, Lill C, Faull RL, Curtis MA, Dragunow M (2014). Global changes in DNA methylation and hydroxymethylation in Alzheimer's disease human brain..

LINK

https://doi.org/10.1016/j.neurobiolaging.2013.11.031

CITATION

Murray HC, Dieriks BV, Swanson MEV, Anekal PV, Turner C, Faull RLM, Belluscio L, Koretsky A, Curtis MA (2020). The unfolded protein response is activated in the olfactory system in Alzheimer's disease..

LINK

https://doi.org/10.1186/s40478-020-00986-7

CITATION

Park TI, Schweder P, Lee K, Dieriks BV, Jung Y, Smyth L, Rustenhoven J, Mee E, Heppner P, Turner C, Curtis MA, Faull RLM, Montgomery JM, Dragunow M (2020). Isolation and culture of functional adult human neurons from neurosurgical brain specimens..

LINK

https://doi.org/10.1093/braincomms/fcaa171

Citations

Dieriks BV, Park TI, Fourie C, Faull RL, Dragunow M, Curtis MA. α-synuclein transfer through tunneling nanotubes occurs in SH-SY5Y cells and primary brain pericytes from Parkinson's disease patients.
https://doi.org/10.1038/srep42984

Stevenson TJ, Murray HC, Turner C, Faull RLM, Dieriks BV, Curtis MA. α-synuclein inclusions are abundant in non-neuronal cells in the anterior olfactory nucleus of the Parkinson's disease olfactory bulb.
https://doi.org/10.1038/s41598-020-63412-x

Murray HC, Dieriks BV, Swanson MEV, Anekal PV, Turner C, Faull RLM, Belluscio L, Koretsky A, Curtis MA. The unfolded protein response is activated in the olfactory system in Alzheimer's disease.
https://doi.org/10.1186/s40478-020-00986-7

Park TI, Schweder P, Lee K, Dieriks BV, Jung Y, Smyth L, Rustenhoven J, Mee E, Heppner P, Turner C, Curtis MA, Faull RLM, Montgomery JM, Dragunow M. Isolation and culture of functional adult human neurons from neurosurgical brain specimens.
https://doi.org/10.1093/braincomms/fcaa171

Gardner B, Dieriks BV, Cameron S, Mendis LHS, Turner C, Faull RLM, Curtis MA. Metal concentrations and distributions in the human olfactory bulb in Parkinson's disease.
https://doi.org/10.1038/s41598-017-10659-6

Coppieters N, Dieriks BV, Lill C, Faull RL, Curtis MA, Dragunow M. Global changes in DNA methylation and hydroxymethylation in Alzheimer's disease human brain.
https://doi.org/10.1016/j.neurobiolaging.2013.11.031

Murray HC, Low VF, Swanson ME, Dieriks BV, Turner C, Faull RL, Curtis MA. Distribution of PSA-NCAM in normal, Alzheimer's and Parkinson's disease human brain.
https://doi.org/10.1016/j.neuroscience.2016.06.003

Highet B, Dieriks BV, Murray HC, Faull RLM, Curtis MA. Huntingtin Aggregates in the Olfactory Bulb in Huntington's Disease.
https://doi.org/10.3389/fnagi.2020.00261

Murray HC, Dieriks BV, Swanson MEV, Anekal PV, Turner C, Faull RLM, Belluscio L, Koretsky A, Curtis MA. The unfolded protein response is activated in the olfactory system in Alzheimer's disease.
https://doi.org/10.1186/s40478-020-00986-7

Murray HC, Swanson MEV, Dieriks BV, Turner C, Faull RLM, Curtis MA. Neurochemical Characterization of PSA-NCAM(+) Cells in the Human Brain and Phenotypic Quantification in Alzheimer's Disease Entorhinal Cortex.
https://doi.org/10.1016/j.neuroscience.2017.12.019

Dieriks BV, Dean JM, Aronica E, Waldvogel HJ, Faull RLM, Curtis MA. Differential Fatty Acid-Binding Protein Expression in Persistent Radial Glia in the Human and Sheep Subventricular Zone.
https://doi.org/10.1159/000487633

Jansson D, Dieriks VB, Rustenhoven J, Smyth LCD, Scotter E, Aalderink M, Feng S, Johnson R, Schweder P, Mee E, Heppner P, Turner C, Curtis M, Faull R, Dragunow M. Cardiac glycosides target barrier inflammation of the vasculature, meninges and choroid plexus.
https://doi.org/10.1038/s42003-021-01787-x

Step 6

Coppieters N, Dieriks BV, Lill C, Faull RL, Curtis MA, Dragunow M. Global changes in DNA methylation and hydroxymethylation in Alzheimer's disease human brain.
https://doi.org/10.1016/j.neurobiolaging.2013.11.031
